# Family-Specialized Transformer for L-cystathionine gamma-lyase Engineering and Its Structural Interpretation

**DOI:** 10.34133/csbj.0073

**Published:** 2026-06-05

**Authors:** Ungyu Lee, Minho Park, Byungkun Song, Nam-Chul Ha

**Affiliations:** ^1^ Department of Agricultural Biotechnology, and Research Institute of Agriculture and Life Sciences, CALS, Seoul National University, Seoul 08826, Republic of Korea.; ^2^ Center for Food and Bioconvergence, Seoul National University, Seoul 08826, Republic of Korea.

## Abstract

The diversity of protein structures and reaction mechanisms complicates general-purpose artificial intelligence models for enzyme engineering, motivating family-specialized models. In this study, we developed EnzFormer, a specialized artificial intelligence pipeline for engineering *Staphylococcus aureus* L-cystathionine gamma-lyase (SaMccB). To overcome the scarcity of experimental labels, we used GPT-4o to generate putative activity labels for cystathionine gamma-lyase homologs, leveraging species-level ecological and evolutionary metadata as a proxy for functional selection. Using these labels, we trained a Transformer classifier on embeddings from the ESM Cambrian protein language model. From an exhaustive single-mutant library, in silico prioritization nominated 4 variants for testing and identified SaMccB V129G with a ~2-fold increase in catalytic turnover relative to the wild type. Val129 is distal to the active site; crystallographic and biochemical analyses suggest that V129G weakens local packing, thereby increasing the conformational flexibility of the active site loop, consistent with faster conformational steps in the catalytic cycle. Together, these results suggest that combining large language model-derived evolutionary priors with a family-specialized predictive model can identify distal mutations that modulate enzyme dynamics.

## Highlights

• LLM-driven ecological priors enable large-scale functional labeling of CGL homologs.
• EnzFormer integrates ESMC embeddings to accurately predict putative enzyme activity.
• In silico screening identifies a distal V129G variant with a 2-fold higher *k*_cat_.
• V129G illustrates a stability–activity trade-off via active site loop flexibility.
• Specialized AI models reveal nonobvious allosteric sites modulating enzyme dynamics.

## Graphical Abstract

**Figure:**
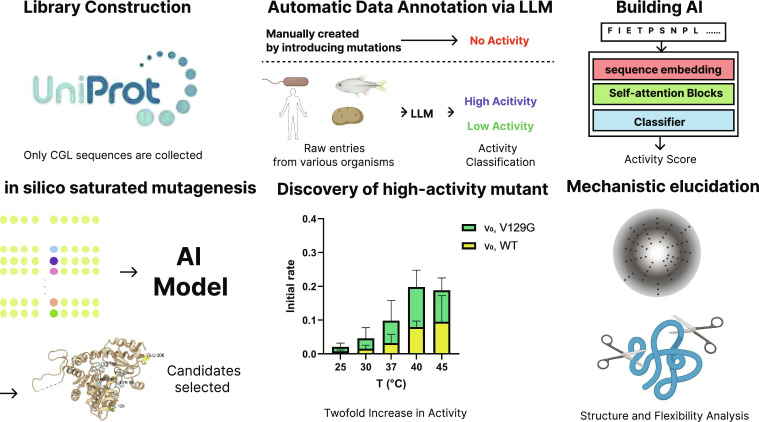

## Introduction

Enzymes are essential biological catalysts with broad utility in industrial, medical, and environmental applications [1–4]. However, further improving many enzymes can be difficult because natural selection may already have optimized key aspects of their function [5,6]. Traditional protein engineering often relies on exhaustive mutational screening, especially for evolutionarily saturated enzymes—requiring substantial resources to identify rare beneficial variants [5–7].

As an efficient alternative approach, Transformer-based protein language models, such as ESM, have revolutionized enzyme engineering by generating high-dimensional sequence embeddings that capture structural and evolutionary constraints [8–13]. By leveraging these embeddings and deep mutational scanning data, supervised learning models can accurately navigate fitness landscapes and reduce experimental burden by predicting advantageous mutations [13–16]. However, the full potential of these predictive approaches is currently hindered by a critical bottleneck: the lack of high-quality, large-scale labeled datasets [13,17].

To address this critical data limitation, large language models (LLMs) have recently emerged as a promising approach [18,19]. Studies suggest that LLMs can generate consistent, domain-informed labels from limited supervision [18,19]. For example, GPT-4 has successfully annotated cell types in single-cell RNA sequencing datasets with accuracy comparable to expert human curators [18]. Furthermore, recent advances such as the scExtract framework show that LLMs can effectively automate and enhance single-cell RNA sequencing data annotation and integration [19]. A recent study further suggests that language-model representations can encode functionally relevant structural information, including signals related to enzymatic binding-site localization [20]. These findings suggest that LLM-based labeling may provide a scalable way to generate putative annotations for downstream modeling [18,19].

Cystathionine gamma-lyases (CGLs) vary widely across species to fulfill distinct physiological demands [21–25]. Although primarily functioning to supply cysteine, CGLs can repurpose their pyridoxal 5ʹ-phosphate (PLP)-dependent catalytic platform for H_2_S formation, driving stress and antibiotic tolerance in pathogens [23–28]. Across species, CGLs exhibit distinct catalytic regimes; for instance, *Thermobifida fusca* utilizes a housekeeping CGL optimized for steady-state metabolic flux, while *Staphylococcus aureus* SaMccB exhibits a high-turnover kinetic profile adapted for rapid, burst-like defensive responses [21,23,29].

In this study, we developed an enzyme-family-specialized artificial intelligence (AI) pipeline for CGL. This integrated framework links scalable LLM-derived labeling to a specialized Transformer model, using SaMccB as a benchmark. Notably, among 4 model-prioritized candidates, we identified the V129G variant of SaMccB, which exhibits a ~2-fold increase in catalytic turnover (*k*_cat_). Structural and biochemical characterization of the V129G variant provides mechanistic context for the observed *K*_M_–*k*_cat_ and activity–stability trade-offs [30].

## Materials and Methods

### Data curation from UniProt and GPT-4o-based putative activity annotation for high- and low-activity groups

Protein sequences annotated with the Enzyme Commission number EC 4.4.1.1 (CGL) were retrieved from the UniProt database, yielding 13,639 entries [31]. Sequence quality was assessed, and duplicate entries were removed, resulting in 12,231 unique sequences. GPT-4o also assigned a putative temperature category, and sequences predicted to derive from thermophilic or psychrophilic organisms (*n* = 1,042) were excluded. The final dataset comprised 11,189 sequences (Fig. S1). Protein sequences were annotated by a custom Python pipeline that parsed UniProt-style FASTA headers, extracted species names (OS = fields), and queried the OpenAI API (model gpt-4o) with a fixed system prompt. For each species, the model assigned putative CGL activity labels (“High activity” or “Low activity”), inferred from species-level metadata, and assigned 1 of 3 temperature categories (thermophilic > 45 °C, mesophilic 15 to 45 °C, and psychrophilic ≤ 15 °C) [32,33]. API calls were made with temperature = 0 and max_tokens = 20 to reduce randomness and standardize outputs. The full GPT-4o system prompt and output label schema used for automated annotation are provided in Fig. S2.

### Artificial generation of the “No activity” group

A total of 1,330 sequences were generated by systematic in silico single-point mutagenesis of high-confidence CGL sequences (UniProt reviewed entries or proteins with protein existence level PE = 1) [31]. For each of 13 representative CGL proteins (UniProt accessions Q8VCN5, Q60HG7, Q58DW2, Q55DV9, Q19QT7, P55216, P32929, P31373, P18757, O69652, F4K5T2, B4SII9, and A0A125YN40), we retrieved UniProt functional annotations and identified all residues annotated as “active site” or “binding site” [31]. Each annotated residue was then substituted in silico with each of the remaining 19 canonical amino acids, while keeping the rest of the sequence unchanged. This procedure yielded 1,330 single-point mutants in total (76 variants for each of Q8VCN5, Q60HG7, Q58DW2, Q55DV9, Q19QT7, P55216, P32929, P31373, and P18757; 114 for O69652; 133 for F4K5T2; 247 for B4SII9; and 152 for A0A125YN40). Because these substitutions were expected to disrupt catalysis, these mutants were operationally assigned to an auxiliary “No activity” penalty group for model training; this set was introduced to discourage prioritization of active site substitutions and was not intended to represent the full diversity of inactive CGL sequence space. In addition, we included 7 variants from a prior internal mutational scan that were classified as nonfunctional in experimental screening and were therefore used as auxiliary “No activity” anchors. These experimentally screened examples were incorporated to complement the in silico-generated mutants with a small set of measured nonfunctional cases (Fig. S3).

### Sequence embedding with ESMC

The ESMC-600M-2024-12 weights were downloaded from the Hugging Face model repository (https://huggingface.co/EvolutionaryScale/esmc-600m-2024-12) to convert the curated amino acid sequence into high-dimensional numerical representations [34]. Per-residue embeddings were generated for each sequence in our final dataset, resulting in embeddings of dimension *L* × 1152, where *L* represents the residue numbers of each sequence.

### PSSM analysis and ΔPSSM calculation

Position-specific scoring matrices (PSSMs) were generated for the wild-type (WT) SaMccB sequence and the single-point mutants by running a PSI-BLAST search against the National Center for Biotechnology Information nonredundant protein database [35]. The search was performed with 3 iterations, an E-value threshold of 0.05, a word size of 3, and a maximum of 500 target sequences. The change in PSSM score (ΔPSSM) for each possible mutation was then calculated as the difference between the PSSM score of the mutant amino acid and that of the WT amino acid at the given position.

### Development of EnzFormer

Precomputed residue-wise ESM Cambrian (ESMC) embeddings were used as fixed inputs during classifier training and were not fine-tuned [34]. The core of the classifier comprised 5 Transformer encoder blocks, each consisting of a multihead self-attention layer with 16 heads, followed by a position-wise feed-forward network with dimensionality expanded by a factor of 4 [36]. Layer normalization and dropout were employed within each block for regularization. After processing through the Transformer blocks, mean pooling was applied along the residue dimension, and the resulting 1 × 1,152 representation was passed through a linear layer to yield the final classification output with an output dimension of 3, corresponding to the putative groups “High activity,” “Low activity,” and “No activity.”

The Optuna framework [37] was employed to optimize the hyperparameters, with the objective function set to maximize the mean cross-validation F1 score. The search space for the optimization was defined as follows: The number of Transformer blocks (num_blocks) was sampled from an integer range of 4 to 10; the learning rate was sampled from a log-uniform distribution between 1 × 10^−5^ and 5 × 10^−3^; the dropout rate was sampled from a uniform range of 0.1 to 0.7; and weight decay was sampled from a log-uniform distribution between 1 × 10^−6^ and 1 × 10^−2^. The number of attention heads (n_head) was chosen from a categorical set of [4,8,16], and the optimizer was selected from [“Adam”, “AdamW”]. Because the putative “High activity” group was the minority group of greatest practical interest, its decision threshold was treated as a tunable hyperparameter (0.3 to 0.9) rather than fixed at 0.5.

The model’s performance and generalizability were evaluated using a stratified 5-fold cross-validation. The focal loss function was employed as the criterion during training [38]**.** For each of the 5 iterations, the model was trained on 4 folds of the data and validated on the remaining fold. An early stopping mechanism was implemented with a patience of 5 epochs, which halted training if the validation F1 score did not show improvement.

### Label permutation and null-model stress test

Only the 11,189 curated natural mesophilic sequences annotated by GPT-4o were used for this stress test; the auxiliary “No activity” group was excluded. Proteins were represented by 1,152-dimensional mean-pooled ESMC embeddings [34]. To validate the GPT-4o-derived labels, Random Forest (500 estimators, max features=sqrt) and XGBoost (300 estimators, max depth=4, learning rate=0.05) classifiers were evaluated using a stratified 5-fold cross-validation scheme (random state=42) [39,40]. Model performance was quantified by Macro-F1 and Matthews correlation coefficient (MCC) averaged across 5 folds.

Null distributions were established using 500 independent label permutations under 3 conditions: global random shuffling, sequence length-restricted shuffling within 10 equal-frequency bins, and dual-restricted shuffling controlling for both length (5 bins) and the first principal component of amino acid composition (5 bins) across 25 strata. Empirical 1-sided *P* values were calculated as *P* = (*b* + 1)/(*B* + 1), where *b* is the number of permutations meeting or exceeding the observed performance and *B* is the total number of permutations (*B*=500).

### Model ablation

Systematic model ablations were evaluated on the 3-group dataset using 5-fold stratified cross-validation, with results aggregated across 3 random seeds (21, 89, and 178). Models were trained in PyTorch using focal loss (gamma=2), per-fold group weighting, and Adam or AdamW optimization with weight decay. The learning rate schedule utilized a 5-step linear warm-up followed by a 0.95 exponential decay per step. Training proceeded for up to 50 epochs with early stopping (patience=5) based on validation Macro-F1. Final predictions were generated via softmax and argmax, rejecting any prediction with a maximum probability below 0.55 (−1 label).

Baseline hyperparameters were optimized via 30 Optuna trials. A linear baseline processed 1 × 1,152 mean-pooled ESMC embeddings through a 0.2 dropout layer (learning rate 1 × 10^−3^, weight decay 5 × 10^−7^). A multilayer perceptron (MLP) baseline processed the same embeddings through 2 512-dimensional hidden layers with a 0.2 dropout rate (learning rate 5 × 10^−4^, weight decay 2 × 10^−6^). Transformer-specific ablations evaluated model depth (0, 1, 3, or 5 encoder blocks) using fixed hyperparameters: batch size 16, learning rate 2 × 10^−4^, dropout 0.1, weight decay 2 × 10^−5^, and 16 attention heads. Self-attention was ablated by replacing attention sublayers with feed-forward-only blocks across a 5-block depth while retaining residual connections. Input granularity was ablated by mean-pooling residue-level inputs into a single 1 × 1,152 sequence-level embedding prior to the model head.

### Performance metrics and latent space visualization

The predictive performance of the final model in classifying putative activity labels was quantified using standard classification metrics, including accuracy, precision, recall, and F1 score, calculated for each fold of the cross-validation. The high-dimensional output embeddings from the putative activity-label classifier were visually inspected by projecting the embeddings into a 2-dimensional space using the Uniform Manifold Approximation and Projection (UMAP) algorithm [41]. The UMAP parameters were fixed as follows: n_neighbors=15, min_dist=0.1, n_components=2.

### Site-directed mutagenesis

The WT SaMccB used for all experiments in this study was derived from *S. aureus*, and all variants were generated from this template. Candidate point mutations identified by in silico screening were introduced into the WT SaMccB gene, which has been used in previous studies [23], by polymerase chain reaction (PCR)-based site-directed mutagenesis. Complementary primers containing the desired mutations were designed using the QuikChange Primer Design Program (Agilent Technologies) and synthesized by Bioneer (Daejeon, Republic of Korea). PCRs (20 μl) contained 1 μl of plasmid template, 1 μl of each primer, 4 μl of TOPsimple PCR DryMIX-nTaq (Enzynomics, Daejeon, Republic of Korea), and nuclease-free water. Thermal cycling consisted of annealing at 65 °C for 30 s and extension at 72 °C for 5 min. PCR products were treated with DpnI (Takara Bio) for 1.5 h and transformed into *Escherichia coli* XL1-Blue competent cells. Plasmids from selected colonies were purified and verified by Sanger sequencing (NICEM, Seoul, Republic of Korea). Primers used for mutagenesis are listed in Table S2.

### Expression and purification of SaMccB proteins

The pET-21a-LIC plasmids containing the WT SaMccB sequence and each mutant sequence were individually transformed into *E. coli* BL21 (DE3) competent cells for recombinant protein expression. A single colony of each transformant was used to inoculate 1.5 L of Luria–Bertani medium supplemented with ampicillin. The cultures were grown at 37 °C with vigorous shaking until the optical density at 600 nm reached approximately 0.6. Protein expression was then induced by the addition of isopropyl-β-d-thiogalactopyranoside to a final concentration of 0.5 mM. Cultures were further incubated for 6 h at 30 °C.

Cells were harvested by centrifugation at 6,290 g for 5 min. The resulting cell pellet was resuspended in 50 ml of lysis buffer (20 mM Hepes and 150 mM NaCl, pH 7.5). Cell disruption was performed on ice using an ultrasonicator for 3 sonication cycles (5 min at 70% amplitude and 5-min resting period). The cell lysate was clarified by centrifugation at 32,310 g for 30 min at 4 °C. The resulting supernatant was subjected to immobilized metal affinity chromatography with Ni-nitrilotriacetic acid resin based on lysis buffer. The Ni-nitrilotriacetic acid resin was then washed with 5 column volumes of wash buffer (20 mM Hepes, 150 mM NaCl, and 20 mM imidazole, pH 7.5). His-tagged SaMccB proteins were eluted with an elution buffer (20 mM Hepes, 150 mM NaCl, and 250 mM imidazole, pH 7.5). The pooled fraction was subjected to a HiTrap Q HP 5-ml anion-exchange column (Cytiva) and then was eluted with a linear NaCl gradient. As a final polishing step, the fractions containing proteins were pooled, concentrated, and subjected to size-exclusion chromatography using a HiLoad 16/600 Superdex 200 pg column (Cytiva) pre-equilibrated with lysis buffer. The purity and molecular weight of purified proteins were confirmed by sodium dodecyl sulfate-polyacrylamide gel electrophoresis analysis.

### Enzyme activity assay

The enzymatic activities of the purified WT and mutant SaMccB proteins were experimentally quantified using a fluorescence-based continuous assay. All measurements were performed in a 96-well plate format using a Varioskan LUX multimode microplate reader (Thermo Fisher Scientific) in a total volume of 200 μl. The reaction mixture consisted of 3 μM purified enzyme and 30 μM PLP cofactor in the assay buffer (20 mM Hepes and 150 mM NaCl, pH 7.5) at the indicated temperatures. The reaction was monitored in the presence of the fluorescent probe 7-azido-4-methylcoumarin (100 μM) at an excitation wavelength of 365 nm and an emission wavelength of 450 nm [*42*].

For general activity comparisons, reactions were initiated by addition of 100 μM L-cysteine. For determination of kinetic parameters (*K*_M_–*k*_cat_), reactions were initiated with L-cysteine across a concentration range of 0.05 to 20 mM (0.05, 0.1, 0.2, 0.5, 1, 2, 5, 10, 15, and 20 mM). Fluorescence was recorded immediately after substrate addition. A 7-amino-4-methylcoumarin standard curve (0 to 50 μM; 0, 2.5, 5, 10, 20, 30, 40, and 50 μM) was measured on the same plate, and fluorescence units were converted to 7-amino-4-methylcoumarin concentration using the calibration curve. Initial velocities were obtained from the linear portion of the progress curves.

### Crystallization, data collection, and structure determination of V129G variant protein

Purified V129G variant protein, in 20 mM Hepes (pH 7.5) and 150 mM NaCl and supplemented with 800 μM PLP, was concentrated to 15 mg/ml and screened for crystallization using the sitting drop vapor diffusion method. Initial crystals were obtained in the PEGION H12 condition (1% w/v tryptone, 0.05 M Hepes sodium [pH 7.0], 20% polyethylene glycol [molecular weight 3,350], 0.01 M sodium azide). Diffraction-quality crystals were optimized using the hanging drop method with a final condition of 1.5% tryptone, 16% polyethylene glycol [molecular weight 3,350], 0.05 M Hepes (pH 7.0), and 0.01 M sodium azide.

For data collection, crystals were briefly soaked (5 s) in mother liquor supplemented with 25% glycerol as a cryoprotectant and flash-cooled in liquid nitrogen. X-ray diffraction data were collected at the PLS-II 7A beamline (Pohang Light Source) and processed and scaled using HKL2000 [43]. The structure was solved by molecular replacement with Phaser [44] within the PHENIX software suite [45], using the WT SaMccB structure (Protein Data Bank [PDB] ID: 6KGZ) [23] as the search model. Iterative cycles of model building and refinement were performed using Coot [46] and phenix.refine [47]. Final refinement statistics are summarized in Table S3. Coordinates and structure factors for V129G have been deposited in the PDB under accession code 9XLU.

To compare the electron density surrounding the flexible loop region (residues 95 to 103) between WT and V129G, both structures were subjected to identical refinement strategies (strategy=individual_sites+individual_adp, optimize_xyz_weight=true, optimize_adp_weight=true) using a 2.3-Å high-resolution cutoff [45]. To minimize model bias, a composite omit map was generated [48,49] using phenix.composite_omit_map. Furthermore, to validate the density of the Tyr99 side chain, a polder omit map was calculated using phenix.polder [50] with the selection “chain A and resi 99 and not name N+CA+C+O”. All electron density maps for analysis were generated using phenix.mtz2map and visualized in PyMOL [51], whereas all other structural representations were prepared using UCSF ChimeraX [52].

### Limited proteolysis

Limited proteolysis assays were conducted in buffer (20 mM Hepes [pH 7.5], 150 mM NaCl, and 2 mM CaCl₂). Purified WT or V129G SaMccB was diluted to a final concentration of 0.3 mg/ml and preincubated with specified concentrations of PLP (0 to 20 μM). Reactions were initiated at room temperature by adding N-tosyl-L-phenylalanine chloromethyl ketone-treated trypsin (Sigma-Aldrich, #T1426) to a final concentration of 0.3 μg/ml (1:1,000 w/w ratio, trypsin:MccB). At the indicated time points, aliquots were removed, quenched with a 6× sodium dodecyl sulfate-polyacrylamide gel electrophoresis loading buffer, and immediately boiled at 95 °C for 5 min. All gels were stained with Coomassie Blue, and band intensities were quantified using ImageJ [53].

### Molecular dynamics simulations

Initial coordinates for the WT and V129G systems were taken from the WT SaMccB structure (PDB ID: 6KGZ) and the V129G crystal structure determined in this study (PDB ID: 9XLU), respectively. All-atom molecular dynamics simulations were performed using GROMACS 2025.3 with the CHARMM36-jul2021 force field and transferable intermolecular potential 3-point (TIP3P) water [54–56]. Standard protonation states appropriate for near-neutral pH were assigned automatically during topology generation with pdb2gmx. Systems were solvated in a dodecahedral box (1.0-nm minimum distance), neutralized, and supplemented with 0.15 M NaCl. Long-range electrostatic interactions were treated with the particle mesh Ewald method using a 1.0-nm cutoff for both Coulombic and van der Waals interactions [57]. All bonds involving hydrogen atoms were constrained using the LINCS algorithm, allowing an integration time step of 2 fs [58]. Energy minimization employed the steepest descent algorithm (emtol = 1,000 kJ/mol/nm; maximum 50,000 steps). Equilibration comprised 100-ps NVT and 100-ps NPT ensembles with positional restraints applied to protein heavy atoms (1,000 kJ mol^−1^ nm^−2^ in each Cartesian direction), maintaining 310 K (v-rescale thermostat, tau_T = 0.1 ps) and 1 bar (c-rescale barostat, tau_P = 2.0 ps) [59,60]. Five independent 100-ns unrestrained production runs were executed using distinct velocity seeds.

Trajectories were processed to remove periodic boundary artifacts, recentered on the protein, and stripped of solvent and ions. The resulting protein-only trajectories were then aligned to a reference structure using Cα atoms from the core region, excluding residues 95 to 103 and 124 to 134 from the least-squares fit to avoid damping motion in the flexible loop and the helix of interest. In addition, snapshots were extracted every 1 ns from 5 independent 100-ns production runs.

To quantify local hydrophobic packing around residue 129, hydrophobic residues were defined as Ala, Val, Ile, Leu, Met, Phe, Tyr, Trp, or Pro. Hydrophobic residues within the helix with residues 124 to 134 were identified based on WT residue identities. To enable direct comparison between WT and V129G, hydrophobic partner residues outside the helix were selected from the WT trajectories as the union of residues whose heavy atoms came within 0.60 nm of heavy atoms in the helix with residues 124 to 134 in the first analyzed frame of each WT replicate. This procedure yielded the partner sets 93, 117, 119, 120, 139, 141, 160, 161, and 166. Contact occupancies for all helix-partner residue pairs were then computed separately for each replicate in both WT and V129G trajectories using the closest heavy-atom distance, with contacts defined by a 0.45-nm cutoff. For each system, replicate-level contact occupancies were averaged across 5 independent trajectories. For frame-wise distribution analysis, the total number of helix-partner contacts present in each frame was calculated by summing all residue-pair contacts and then pooled across the 5 replicates of each system.

### Analysis of EnzFormer performance under sequence-identity-aware and species-holdout splits

For sequence-identity-aware evaluation, we excluded the 1,330 in silico mutation-bearing auxiliary “No activity” sequences and performed clustering and leakage analysis on the remaining 11,189 sequences [61]. The effectiveness of the sequence-aware partitioning was validated by quantifying the maximum global sequence identity between validation and training sets. For each validation sequence, candidate training neighbors were first identified via 3-mer term frequency-inverse document frequency cosine distance, followed by precise global alignment using Biopython pairwise2.align.globalms to calculate the nearest-train identity [62,63].

Targeted hyperparameter retuning was conducted for each split scheme to ensure that performance variations were not biased by suboptimal configurations. The search space encompassed 6 experimental conditions, including variations in learning rate (1 × 10^−4^ to 5 × 10^−4^), dropout (0.2), and weight decay (1 × 10^−5^ to 5 × 10^−5^). Final configurations for each split were selected based on the 5-fold mean Macro-F1 score as the primary metric. Sequence-identity-aware analysis was performed as a robustness evaluation rather than as the primary model-selection criterion. Because the intended prospective application was within-family ranking in a homolog-rich 7,220-member SaMccB single-point mutant library, the random-split model was used for final prospective prioritization.

For species-holdout and genus-holdout evaluation, sequences were grouped using taxonomic labels parsed from the FASTA header OS= field. Species-holdout grouping was based on the exact species name, whereas genus-holdout grouping was based on the corresponding genus annotation derived from the same field. After removing all auxiliary “No activity” samples, 11,189 natural sequences were retained for grouped 5-fold cross-validation. In each setting, all sequences belonging to the same species or genus were assigned to a single fold, such that no species or genus overlapped between training and validation folds, respectively. Fold assignment was generated with a fixed random seed of 42 using a constrained grouped k-fold optimization procedure that approximately balanced fold size and class composition across folds. Because these analyses were performed as additional robustness checks, we did not perform separate hyperparameter retuning for the species-holdout or genus-holdout splits.

### Benchmarking EnzFormer

As a small independent experimental benchmark, we manually curated steady-state *k*_cat_ measurements for *Saccharomyces cerevisiae* CGL variants [64]. This benchmark consists of 10 samples, comprising 1 WT and 9 variant sequences, all characterized under identical experimental conditions. A conservative threshold of 0.8 was applied to specifically exclude sequences highly homologous to the benchmark set. This criterion ensured the independence of the evaluation while maintaining the scale of the training data, resulting in the exclusion of approximately 1% (110 records) of the original dataset.

For a comprehensive performance evaluation, EnzFormer was compared against both sequence-only and sequence-plus-substrate models. Sequence-only baselines included ESM2 zero-shot scores [11], calculated as the sum of log-probability differences between mutant and WT residues following the procedure described by previous research [65]. For substrate-aware models (CataPro, UniKP, and DLKcat) [16,66,67], simplified molecular input line entry system information corresponding to the assay conditions was integrated. Ranking scores were defined as the predicted log10 (*k*_cat_) for CataPro and UniKP, and the log-transformed positive predictions for DLKcat. In the case of EnzFormer, the putative “High activity” group probability, averaged across 5 fold-specific checkpoints, was utilized as the final ranking score. All gold-label *k*_cat_ values were log10-transformed prior to statistical analysis for normalization. The predictive performance of each model was quantified by calculating Spearman’s ρ and Kendall’s τ correlation coefficients between the measured log10 (*k*_cat_) and the model-generated scores.

### Residue-level attribution analysis

Residue-level attribution analysis was performed for 5 representative sequences (WT, V129G, H284D, H284Y, and E306Y). For each sequence, the same precomputed embedding tensor used for EnzFormer inference was used as input to the trained ensemble models. Integrated gradients (IGs) were calculated with Captum using a zero-embedding baseline and 50 interpolation steps [68,69]. IG was computed with respect to the ensemble-predicted class, and attribution tensors were averaged across ensemble members. Residue-level IG scores were defined as the L2 norm of the attribution vector across embedding dimensions.

Residue-level attention scores were calculated from the same ensemble outputs. Attention tensors were extracted during inference, averaged across ensemble members, transformer blocks, and attention heads, and reduced to a residue-by-residue attention matrix. Diagonal elements were set to zero, and an incoming-attention score for each residue was calculated as the sum of off-diagonal attention weights received from all other residues.

### Relationship between LLM-derived labels and pairwise sequence similarity

To assess whether the GPT-derived activity labels could be explained primarily by simple sequence similarity, we performed a pairwise sequence-similarity analysis using the curated training dataset. Only the GPT-derived high-activity and low-activity classes were included in the primary analysis, whereas the “No activity” class was excluded because it was not generated by the same LLM-labeling procedure. This yielded 11,189 labeled sequences for analysis.

A total of 20,000 unique unordered sequence pairs were sampled uniformly without replacement. Self-pairs and duplicated unordered pairs were excluded. Exact identity was defined as the number of matched aligned residues divided by the aligned sequence length. Each pair was annotated as either same-label or different-label and further assigned to 1 of 3 pair types: high–high, low–low, or high–low.

To quantify the relationship between sequence similarity and label agreement, we compared the exact-identity distributions between same-label and different-label pairs and across the 3 pair types. Three summary statistics were calculated: area under the receiver-operating characteristic curve (AUC) for predicting same-label status from exact sequence identity, the median difference in exact identity between same-label and different-label pairs, and the rank-biserial correlation as a rank-based effect-size measure. Confidence intervals were estimated by bootstrap resampling with replacement (1,000 replicates).

To assess whether the observed association exceeded a random-label baseline, a label-permutation test was performed on the same fixed pair table. Labels were randomly permuted across sequence IDs while preserving the precomputed sequence-similarity values, after which same-label/different-label assignments and all 3 summary statistics were recalculated. This procedure was repeated 1,000 times to generate null distributions, and 2-sided permutation *P* values were calculated accordingly.

## Results

### LLM-based data labeling

**Fig. 1.**
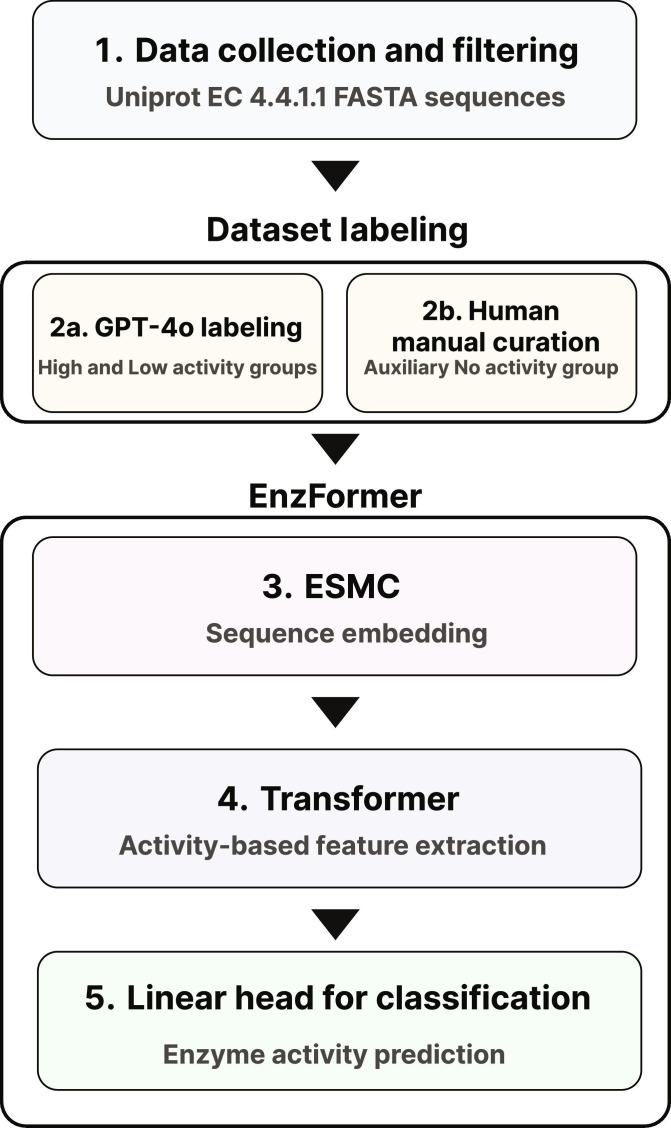
A schematic representation of the cystathionine gamma-lyase (CGL) enzyme activity prediction pipeline. The workflow consists of 3 main stages: (1) data collection and filtering, (2) sequence labeling, and (3 to 5) EnzFormer module for the activity prediction. (1) Data Collection and Filtering: CGL-related protein sequences were retrieved from UniProt (EC 4.4.1.1) and curated for model input. (2) Sequence Labeling: 2a. Sequences were automatically annotated into putative high- and low-activity groups using GPT-4o. 2b. A third putative “No activity” group was manually created by introducing mutations into catalytically essential residues. (3) Sequence Embedding (ESM Cambrian [ESMC]): Each sequence was encoded using ESMC model, producing structure- and evolution-aware embeddings. (4) Feature Extraction (Transformer): A Transformer encoder was applied to extract activity-relevant features from the ESMC embeddings. (5) Classification: A final linear classification head predicted putative enzyme activity groups, enabling in silico prioritization of functional variants.

To integrate scalable LLM-derived putative activity labeling with a specialized Transformer-based classification model, we established an enzyme-family-specialized pipeline comprising 3 primary stages: GPT-4o-based putative activity annotation, EnzFormer training, and in silico mutation prioritization (Fig. 1). Specifically, for the initial stage, we implemented a high-throughput annotation strategy using GPT-4o to assign putative labels based on species-level metadata [70].

**Fig. 2.**
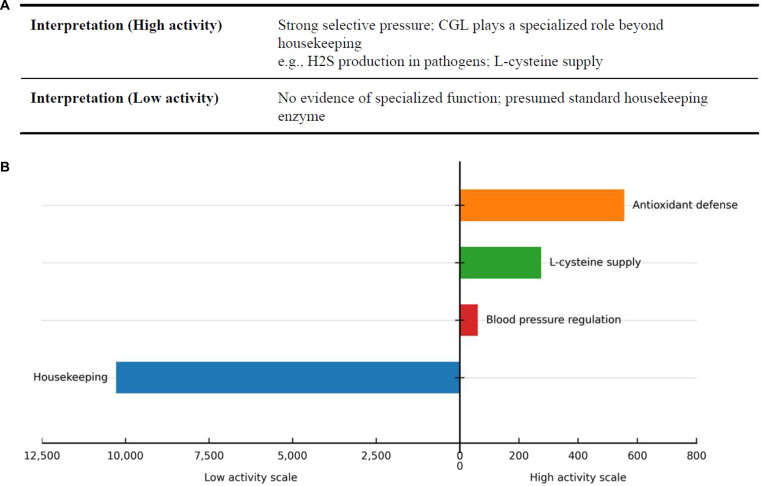
The GPT-4o-guided sequence labeling step. (A) Core prompt for GPT-4o-guided sequence labeling. For clarity, only the essential components of the prompt used for activity classification are displayed in this figure; the full verbatim system prompt and output label schema are provided in Fig. S2. This prompt is integrated into a Python script (gpt_labeling.py) designed to automate the sorting of sequences into putative high- and low-activity groups. (B) Functional categorization of low- and high-activity groups. The bar chart illustrates the distribution of functional categories according to putative activity labels. The left panel (blue) represents the “Low activity” (Housekeeping) group, showing a predominant enrichment of housekeeping functions. The right panel displays the “High activity” group, in which distinct biological functions are highlighted, including antioxidant defense (orange), L-cysteine supply (green), and blood pressure regulation (red). The x-axis indicates the relative activity scale, with error bars denoting SDs.

As illustrated in Fig. 2A, a fixed GPT-4o system prompt was used to assign putative activity labels to each of the 12,231 sequences, guiding the model to classify them into putative “high activity” and “low activity” categories based on inferred organismal and ecological constraints. “High activity” proteins were defined as those under strong selective pressure in specific contexts, such as H_2_S production in host-associated bacteria for antibiotic tolerance [26,28,71] or endogenous L-cysteine supply during dietary restriction [25], whereas “low activity” proteins represented those with general housekeeping roles. By embedding these ecological contexts into the prompt as few-shot examples, we aimed to improve labeling consistency and to help GPT-4o assign putative labels in a more standardized manner.

We observed a persistent tendency for unconstrained models to erroneously recommend mutations at catalytically essential residues in our previous work. To mitigate this, we intentionally generated the “No activity” group by mutating these active and binding sites to act as a penalty group, explicitly teaching the model not to prioritize mutations at the conserved residues at the active and substrate binding regions. The auxiliary “No activity” group comprised 1,337 putatively nonfunctional examples. This set included 1,330 sequences generated by systematic in silico substitution of essential substrate-binding residues in high-confidence CGL sequences [31].

Initially, we developed a preliminary version of our model, which was trained to classify enzyme groups based on standard EC numbers rather than LLM-derived ecological priors. Utilizing the preliminary model, we identified 7 distal mutations in SaMccB that were predicted to enhance activity. However, experimental validation revealed that all 7 variants exhibited no functional activity (Fig. S3). Consequently, these variants were categorized into the “No activity” group, serving as experimental anchors for the nonfunctional sequence space.

Following the initial annotation, we applied an additional filtering criterion to exclude 1,042 sequences identified by GPT-4o as thermophilic or psychrophilic, ensuring that the dataset was tailored to standard mesophilic conditions. After removing a total of 2,450 sequences throughout the curation pipeline, we obtained 11,189 curated mesophilic sequences. Supplementing these with the auxiliary “No activity” sequences yielded a final dataset of 12,526 sequences, comprising 916 putative “High activity”, 10,273 putative “Low activity”, and 1,337 “No activity” sequences (Fig. S1).

### Validation of LLM-based data labeling

To assess whether the GPT-4o-generated labels contained nonrandom sequence-associated signal, we focused exclusively on the putative “High activity” and “Low activity” groups. We compared the performance of Random Forest and XGBoost classifiers against permutation-based null models with randomly shuffled labels [39,40,70]. While classifiers trained on these randomized labels converged to near-chance levels (Macro-F1 ≈ 0.48, MCC ≈ 0), those trained on the actual GPT-4o-derived labels achieved markedly higher performance, reaching a Macro-F1 of 0.805 and an MCC of 0.642 (Fig. S4). Notably, none of the permuted runs approached the observed scores, suggesting that the LLM-derived labels capture nonrandom information reflected in the ESMC embedding space. These results further indicate that the observed signal is not explained solely by sequence length or bulk amino acid composition and support the use of these labels for downstream modeling within the CGL family.

To further assess whether the LLM-derived labels could be attributed to simple sequence similarity, we performed a pairwise sequence-identity analysis between the putative “High activity” and “Low activity” groups. Using 11,189 labeled sequences, we sampled 20,000 unique unordered sequence pairs and calculated the exact global sequence identity for each pair. Same-label pairs (high–high and low–low) exhibited only a marginally higher median identity than different-label pairs (high–low; 0.161 versus 0.157; delta median = 0.0043) (Fig. S5A). Consistent with this, sequence identity alone yielded near-chance accuracy in predicting label agreement (AUC = 0.5035; rank-biserial correlation = 0.0070). Moreover, the observed AUC did not significantly deviate from the label-permuted null distribution (permutation mean AUC = 0.4999; *P* = 0.595) (Fig. S5B). Together, these results demonstrate that the GPT-4o-derived labels cannot be explained by simple sequence similarity alone.

We then analyzed the patterns and rationales behind GPT-4o’s classifications to verify that our prompting strategy was effective. The putative “High activity” designations were frequently assigned to genera containing well-established pathogens, such as *Mycobacterium* and *Xanthomonas*, where CGL activity has been implicated in virulence and function [27,72,73]. In contrast, some bacteria show low CGL activity due to limited selective pressures. For instance, the soil and freshwater bacterium *Pseudomonas putida* induces the trans-sulfuration pathway exclusively under sulfur-limiting conditions, maintaining only basal expression levels of CGL under standard growth conditions [74]. Similarly, *Bacillus subtilis*, a commonly used laboratory model organism, demonstrates low selective pressure for robust CGL activity; its PatB enzyme (C-S lyase) can partially substitute for MetC despite its relatively high *K*_M_, indicating that high CGL activity is not essential for survival under typical circumstances [75].

Furthermore, an examination of the functional justifications provided by GPT-4o revealed that its decisions were based on biological principles that aligned with our prompt design. The most common reasons cited for high activity, including antioxidant defense and endogenous L-cysteine supply, directly reflect the selective pressures we aimed to identify (Fig. 2B).

### Architecture and training of the Transformer-based activity prediction model

We developed a deep learning model, termed EnzFormer, to predict putative enzyme activity labels by integrating ESMC embeddings with a Transformer-based architecture. EnzFormer leverages fixed, high-dimensional representations generated by the pretrained ESMC protein language model [34], which is a state-of-the-art masked language model. Building upon these embeddings, the Transformer architecture was selected for its self-attention mechanism [36].

**Fig. 3.**
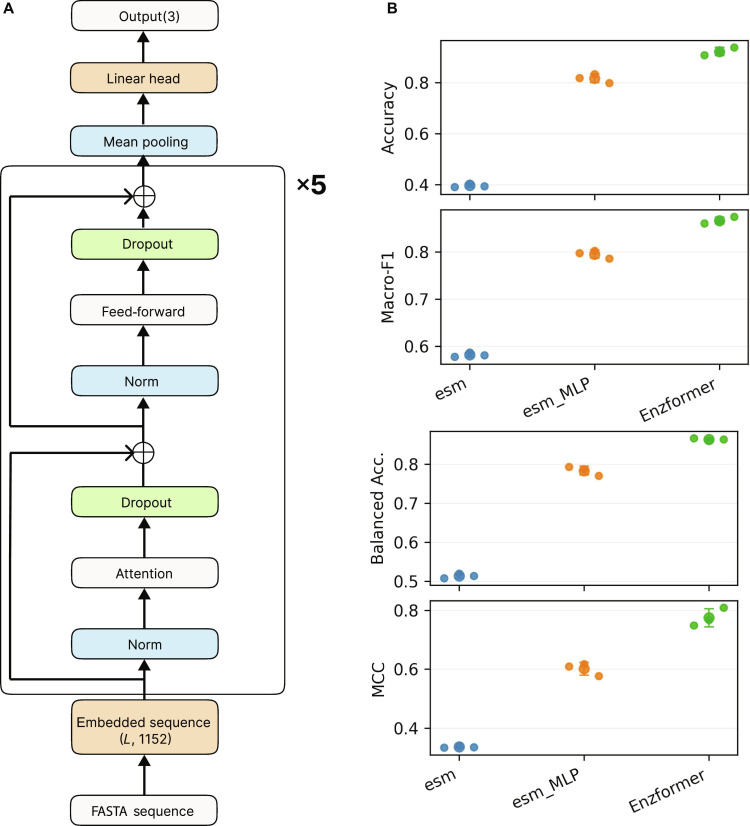
Overall architecture and performance validation of EnzFormer. (A) The model processes protein sequences from FASTA files by first generating embeddings using the ESM Cambrian [ESMC] model with a dimension of *L* × 1,152 (where *L* stands for the protein length). These embeddings are passed through a Transformer encoder composed of 5 stacked blocks (×5). Each block consists of 2 sublayers: attention and feed-forward. The attention sublayer comprises layer normalization (Norm), self-attention mechanism (Attention), and dropout for regularization. The following feed-forward sublayer consists of normalization layer, a feed-forward network, and a dropout layer. Residual connections are applied after both the attention and feed-forward sublayers. After mean pooling, the encoded representation is then fed into a linear classification head to produce an output of 3 putative groups (“High activity”, “Low activity”, and “No activity”). (B) Architectural ablation of EnzFormer. The predictive performances of 3 distinct classifiers—a linear head (esm), a multilayer perceptron (MLP) head (esm_MLP), and the Transformer-based EnzFormer—were compared on the 3-group dataset. For each model, Accuracy, Macro-F1, Balanced Accuracy, and Matthews correlation coefficient (MCC) are presented, with individual points representing separate experimental runs to illustrate performance consistency.

Specifically, each input FASTA sequence is first embedded into a sequence of length *L*, where each amino acid is represented as a 1,152-dimensional vector using the ESMC 600M embedding model. These sequence embeddings are subsequently passed through a Transformer encoder comprising 5 stacked blocks. Each block includes multihead self-attention layers (16 attention heads each block) and a 4-fold feed-forward network. The output embeddings from the final Transformer block are aggregated and fed into a linear classification layer to assign 1 of 3 putative activity labels: High activity, Low activity, or No activity (Fig. 3A).

To determine whether the Transformer module is essential for capturing task-specific information, we conducted an ablation study comparing the full EnzFormer architecture against 2 architectural variants: one with the Transformer module entirely removed and another where it was replaced by an MLP. Our results show that the baseline model, which simply applies mean pooling to raw ESM embeddings with a linear head, performed near chance (Macro-F1 ≈ 0.58). Interestingly, replacing the Transformer with an MLP to increase model depth significantly improved performance (Macro-F1 ≈ 0.80); however, it still failed to match the full EnzFormer (Macro-F1 = 0.867; MCC = 0.775) (Fig. 3B and Figs. S6 and S7). This gap indicates that the performance gain is not explained by increased model depth alone and instead depends on residue-level contextualization enabled by self-attention.

To dissect the Transformer’s contribution, we performed architectural ablations (Figs. S6 to S8). Removing Transformer blocks dropped performance to Macro-F1 ≈ 0.70, but a single block was sufficient to recover nearly full predictive power (Macro-F1 ≈ 0.87), with negligible gains from additional depth. Conversely, removing the attention mechanism or prepooling inputs into a single vector substantially degraded performance. These results confirm that EnzFormer’s advantage stems from residue-level contextualization, providing the necessary precision for in silico prioritization of SaMccB mutants.

### Evaluation of the EnzFormer activity prediction model

**Fig. 4.**
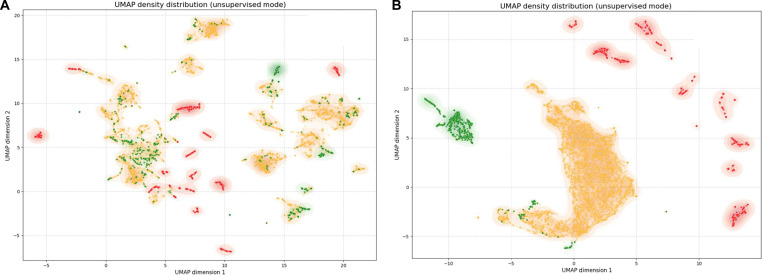
Uniform Manifold Approximation and Projection (UMAP) projection of cystathionine gamma-lyase (CGL) sequences by putative activity groups. (A) UMAP embedding of CGL enzyme sequences generated using the ESM Cambrian [ESMC] model. Sequences are grouped according to putative activity groups: Group 0 (“High activity”, green), Group 1 (“Low activity”, red), and Group 2 (“No activity”, orange). The plot illustrates their distribution without supervised refinement. (B) UMAP embedding after Transformer-based learning: The same CGL sequences after processing through EnzFormer. Groups 0 (“High activity”) and 1 (“Low activity”) exhibit clearer separation, while Group 2 (“No activity”) demonstrates a distinctly altered distribution.

To examine how the learned representations separated the putative activity groups, we employed UMAP [41]. To specifically examine the contribution of the Transformer encoder within EnzFormer, we initially visualized the raw ESMC embeddings of all CGL sequences without the Transformer layers. Although the resulting UMAP projection revealed weak clustering of putative activity groups, considerable overlap remained in the latent space, particularly between the “High activity” (Group 0) and “Low activity” (Group 1) groups (Fig. 4A). The “No activity” group (Group 2) was broadly scattered and showed no discernible clustering, indicating that the pretrained ESMC embeddings alone did not yield clear separation in this UMAP projection.

However, the resulting visualization of the complete EnzFormer revealed a marked improvement in group separability (Fig. 4B). In particular, the “High activity” and “Low activity” groups formed distinct and well-separated clusters, indicating effective feature learning. Although the “No activity” group did not form a clear cluster, its members were distributed more diffusely across the space, unlike the more cohesive “High activity” and “Low activity” groups. These UMAP visualizations qualitatively suggest that EnzFormer reorganized the latent space, thereby improving the separation between the putative “High activity” and “Low activity” groups. To evaluate the predictive accuracy of EnzFormer, we utilized *k*_cat_ measurements of *Saccharomyces cerevisiae* CGL variants as a gold-standard benchmark [64]. Critically, to prevent data leakage, we retrained EnzFormer on a dataset from which all sequences sharing more than 0.8 sequence identity with the *S. cerevisiae* CGL were strictly excluded. This retrained model was then benchmarked against baseline methods (PSSM, ESM2 zero-shot, CataPro, UniKP, and DLKcat), where EnzFormer demonstrated superior performance in both Spearman’s ρ and Kendall’s τ (Fig. S9).

We next examined the generalizability of EnzFormer under stricter controls designed to mitigate both homology- and taxonomy-level leakage. The model was evaluated using partitions restricted to a maximum pairwise sequence identity of 70% and 40% (SeqID70 and SeqID40) between training and test sets (Fig. S10), as well as under grouped 5-fold species- and genus-holdout settings (Fig. S11). Although performance decreased relative to the random split, the persistence of nontrivial signals across all settings argues against the model acting merely as a taxonomic classifier or relying solely on closely related homologs within the CGL family. Thus, our findings confirm that EnzFormer captures fundamental biochemical features rather than simple taxonomic patterns, thereby proving its genuine generalizability.

To verify that the auxiliary “No activity” group did not introduce biased negative clustering, we retrained EnzFormer without the “No activity” group sequences. The average F1 score for natural labels was consistently maintained across the random split as well as the SeqID70 and SeqID40 retraining regimes. This indicates that the primary predictive signal for natural activity levels is robustly preserved and does not depend on the presence of the “No activity” set (Fig. S12).

### EnzFormer-guided enhancement of CGL activity

To demonstrate EnzFormer’s ability to identify high-activity variants, we conducted a proof-of-concept screen of SaMccB single mutants. We constructed a library of 7,220 single-point mutants on SaMccB covering all nonsynonymous substitutions and ranked each variant based on its EnzFormer score. Because EnzFormer does not explicitly account for structural stability, we did not combine EnzFormer and ΔPSSM into a weighted composite score; instead, we first applied a PSSM-based conservativeness filter to deprioritize highly nonconservative substitutions and reduce the risk of poor expressibility [76,77]. The overall mean ΔPSSM across the full single-mutant library was approximately −6.7, and we therefore used ΔPSSM ≥ −7, a cutoff close to the library-wide average, to define the primary follow-up pool. Within this filtered set, variants were prioritized by EnzFormer high-activity probability, from which E306Y, H284Y, and V129G were selected as high-ranking candidates for experimental testing. One additional variant, H284D (ΔPSSM = −9), was included as a single high-risk, model-prioritized exception despite failing the ΔPSSM filter. A brief sensitivity analysis showed that this prioritization was broadly stable to modest relaxation of the ΔPSSM cutoff (Table S8). Using this stepwise procedure, the 4 variants selected for experimental testing were E306Y, H284D, H284Y, and V129G.

To further examine whether EnzFormer’s predictions were associated with specific residues, we analyzed residue-level IGs and attention scores for WT and the 4 selected variants. In several cases, the substituted residue itself became relatively salient in the attribution maps, including residues 129, 284, and 306 in V129G, H284D/H284Y, and E306Y, respectively (Table S6). Attribution was also repeatedly concentrated in a nonrandom subset of residues across samples, with positions such as 380, 304, 350, 35, 17, and 110 consistently appearing among the top-ranked residues by both IGs and attention across WT, V129G, H284Y, H284D, and E306Y (Table S7). These highly attributed positions did not consistently overlap with canonical catalytic residues, which was not unexpected because catalytic residues are strongly conserved within the CGL family. Accordingly, we interpret these attribution analyses as supportive evidence that EnzFormer’s variant prioritization is linked to specific residue-level features, rather than as direct mechanistic proof.

We then conducted the enzyme activity assay with the 4 candidate mutations: E306Y, H284D, H284Y, and V129G. Each variant was expressed in *E. coli*; WT SaMccB and mutants V129G and H284Y showed successful expression, whereas mutants E306Y and H284D failed to yield detectable protein. Consequently, only the successfully expressed V129G and H284Y variants were selected for further purification and quantitative enzymatic activity assays, alongside WT SaMccB as a control.

**Fig. 5.**
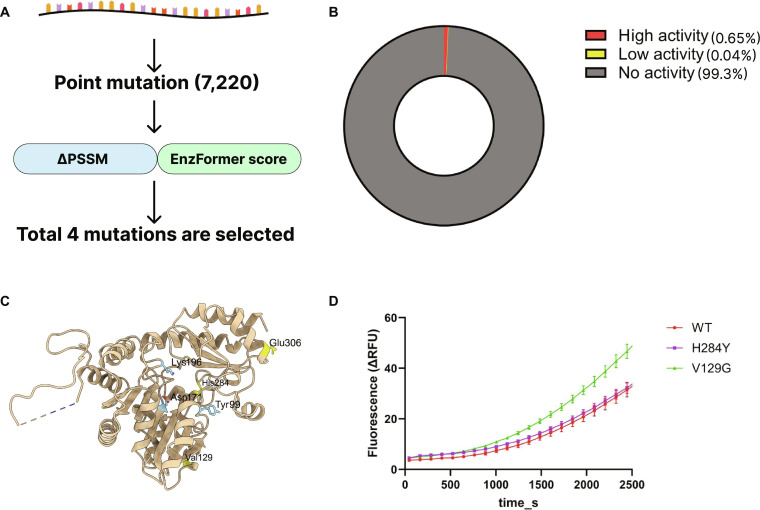
In silico screening using EnzFormer and experimental analysis of the mutants. (A) Workflow for prioritizing candidate beneficial single-point mutations in wild-type (WT) SaMccB. The WT SaMccB cystathionine gamma-lyase (CGL) sequence was used to generate 7,220 single-point mutants. Each variant was evaluated using the change in position-specific scoring matrix score (ΔPSSM) (≥ −7) and EnzFormer scores, and top 4 mutations with favorable predicted effects were selected for further analysis. (B) Donut chart depicting the AI model-based classification of single-point mutations into 3 putative activity categories: “High activity” (0.65%), “Low activity” (0.04%), and “No activity” (99.3%). (C) Structural representation of CGL highlighting pyridoxal 5ʹ-phosphate (PLP)-binding residues (Y99, D171, and K196; sky blue) and targeted single-point mutation sites (V129, H284, and E306; yellow) examined in this study. (D) Time-dependent fluorescence traces of WT, H284Y, and V129G measured at 37 °C. Each variant was measured with 4 biological replicates (*n* = 4), and all signals represent blank-subtracted fluorescence and are shown as means ± standard error of the mean (SEM). WT, H284Y, and V129G traces are displayed in red, green, and purple, respectively. V129G exhibited a faster rate of increase than WT, whereas H284Y showed a nonsignificant increase. ΔRFU, change in relative fluorescence units.

The enzymatic activity of SaMccB was quantified by monitoring the fluorescence generated from the reaction between H₂S and 7-azido-4-methylcoumarin in the assay buffer. Using this assay, we observed that the V129G variant showed higher catalytic activity than WT SaMccB under the assay conditions, whereas H284Y showed activity comparable to WT (Fig. 5D).

### Crystal structure and increased B-factors of the V129G variant

**Fig. 6.**
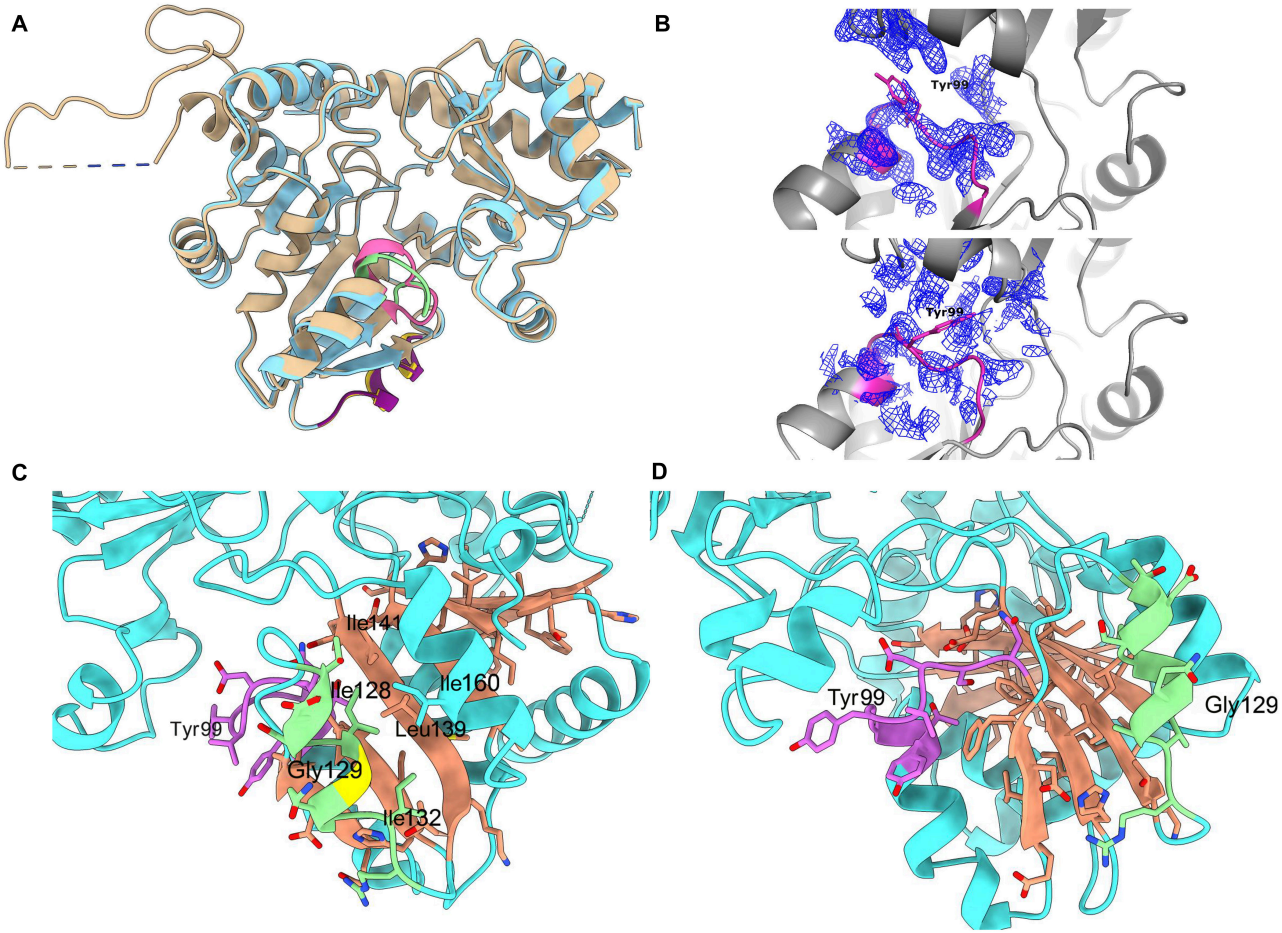
Structural analysis of the V129G variant. (A) Cartoon representation and alignment of wild-type (WT) SaMccB (salmon, PDB ID 6KGZ) and V129G (sky blue). In the WT structure, residues 95 to 103 are highlighted in pink and residues 124 to 134 are highlighted in yellow. In the V129G structure, residues 95 to 103 are highlighted in green and residues 124 to 134 are highlighted in purple. (B) 2mFo–DFc electron density maps (contoured at 1.0 σ) surrounding the active site loop (residues 95 to 103, shown in magenta). WT SaMccB (top). V129G (bottom). (C and D) Cartoon representation of WT SaMccB (salmon) and V129G (sky blue) in different viewpoints. Residues 95 to 103 are highlighted in pink, and residues 124 to 134 are highlighted in green, and the intervening β-sheet is shown in orange.

To investigate the mechanism by which the V129G mutation increases enzymatic activity, we determined the crystal structure of the V129G variant at 2.33-Å resolution (Table S3). Structural alignment with the apo WT SaMccB (PDB ID: 6KGZ) revealed a nearly identical overall fold, with a Cα root-mean-square deviation of 0.291 Å (Fig. 6A). Despite the high overall similarity, a notable difference was observed in a critical active site loop (residues 95 to 103) that contains the essential PLP-binding residue, Tyr99. In the V129G structure, the electron density for this entire loop, including Tyr99, was poorly defined in a different conformation (Fig. 6B).

In both the WT and the V129G variant structures, the 129th residue is located in the middle of a short α-helix (residues 124 to 134, green in Fig. 6C), which interacts with the central hydrophobic β-sheet (orange in Fig. 6C). The short α-helix containing Val129 and the active site loop containing Tyr99 (residues 95 to 103; pink in Fig. 6C) are positioned across the central hydrophobic β-sheet. The V129G substitution maintains the α-helical conformation but removes the partially solvent-exposed valine side chain (Fig. 6C). Consistent with this structural alteration, molecular dynamics simulations showed a significant overall reduction in hydrophobic-contact occupancy in V129G relative to WT. Across the 45 hydrophobic contacts analyzed, the overall mean occupancy was lower in V129G than in WT (0.1724 versus 0.1906; Δ = −0.0182, −9.6%; trajectory-level exact permutation p = 0.0159). The Val129–Ile160 interaction likewise changed from a near-persistent contact in WT to a more intermittent one in V129G (0.9257 versus 0.6723; Δ = −0.2534, ~27% decrease across trajectories), although individual contact-level differences did not remain significant after multiple-testing correction (Fig. S13 and Table S5). The V129G variant exhibited a higher average Cα B-factor (35 Å^2^) across the entire chain compared to the WT (31 Å^2^), consistent with increased flexibility. This effect was especially pronounced locally, affecting both the Tyr99-containing loop and the Gly129-containing α-helix. Specifically, the average B-factor for the disordered active site loop containing Tyr99 (residues 95 to 103) increased dramatically from 42 Å^2^ (WT) to 61 Å^2^ (V129G), and for the α-helix encompassing the V129G substitution, it rose from 31 to 42 Å^2^ (Table S4).

### V129G variant SaMccB protein has greater structural flexibility

**Fig. 7.**
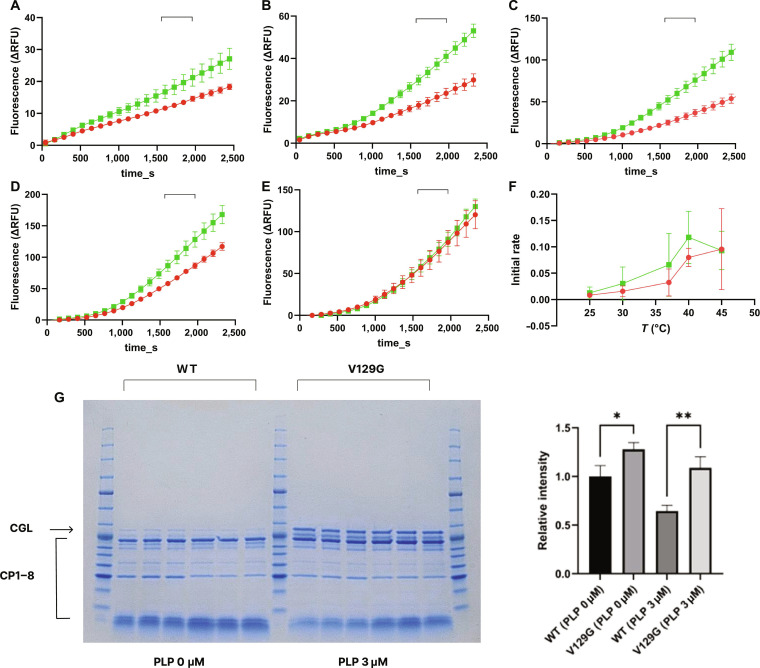
Temperature-dependent activity profiles of wild-type (WT) and V129G cystathionine gamma-lyase (CGL). (A to E) Time-dependent fluorescence traces of WT and V129G CGL measured at 25 °C (A), 30 °C (B), 37 °C (C), 40 °C (D), and 45 °C (E). Each condition was measured with 6 biological replicates (*n* = 6), and all signals represent blank-subtracted fluorescence. Data are shown as means ± standard error of the mean (SEM). WT and V129G traces are displayed in red and green, respectively. The linear region used for rate determination (1,620 to 1,905 s) is indicated by a horizontally clipped rectangular marker. (F) Initial velocities (*V*_0_), calculated from the linear regions of the fluorescence trajectories at each temperature, plotted for WT (red) and V129G (green). The plot highlights the enhanced activity of V129G at 25 to 40 °C and the loss of this gain of function at 45 °C. (G) Limited proteolysis assay of WT and V129G. Representative sodium dodecyl sulfate-polyacrylamide gel electrophoresis (SDS-PAGE) gel (left) shows limited proteolysis of WT and V129G proteins at 80 min in the apo (0 μM pyridoxal 5ʹ-phosphate [PLP]) and holo (3 μM PLP) states, and quantification of the major low-molecular-weight degradation product (right, *n* = 3). Data are normalized to the WT apo state and presented as means ± SD. Statistical significance was determined by 1-way analysis of variance (ANOVA) followed by Tukey’s multiple comparison test (*P < 0.05, **P < 0.01). CGL and CP1–8 indicate the full-length CGL and its major cleavage products, respectively. ΔRFU, change in relative fluorescence units.

To analyze the temperature-dependent activity of the enzymes, we measured the enzymatic activities over a range of 25 to 45 °C. Within its stable operating range of 25 to 40 °C, the variant substantially outperformed the WT enzyme, demonstrating an approximate 50% increase in activity at 25 °C and a nearly 2-fold improvement at 30 and 37 °C. However, the functional stability of V129G was compromised at 45 °C, where its activity dropped to match WT levels, indicating a vulnerability to thermal stress that abolishes its catalytic advantage (Fig. 7).

To directly probe conformational dynamics in solution, we performed limited proteolysis assays. The V129G variant displayed a distinct digestion pattern with higher sensitivity to proteolysis compared to the WT enzyme. Quantitative analysis of triplicate measurements confirmed this observation, revealing a statistically significant increase in proteolytic susceptibility for V129G (Fig. 7G and Fig. S14).

These biochemical data are consistent with our crystallographic and computational observations. As shown in Fig. 6, the V129G structure is characterized by less defined electron density and increased local B-factors both in the vicinity of the mutation and the active site loop containing Tyr99. Collectively, the increased proteolytic susceptibility and elevated crystallographic B-factors support the interpretation that V129G increases local conformational flexibility.

### Enhanced catalytic turnover and kinetic trade-offs of the V129G variant

**Fig. 8.**
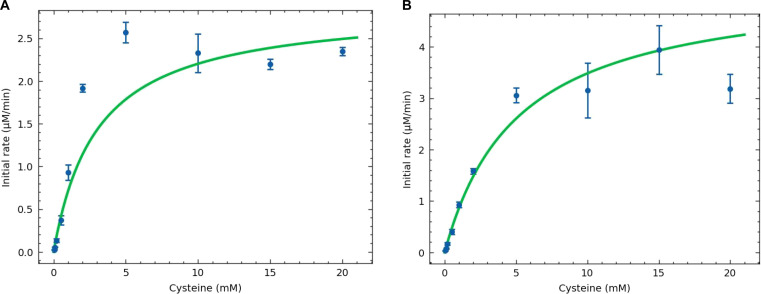
Steady-state kinetics for wild-type (WT) and V129G enzymes. (A and B) Michaelis–Menten fits of initial velocities measured over a range of L-cysteine concentrations for the WT enzyme (A) and the V129G variant (B).

Kinetic analysis revealed that the V129G variant exhibited an approximately 2-fold increase in both maximum velocity (*V*_max_ = 5.27 versus 2.87 μM/min) and turnover number (*k*_cat_ = 0.0293 versus 0.0160 s^−1^) compared to the WT enzyme. This enhanced catalytic rate was accompanied by a moderate decrease in substrate affinity, with the *K*_M_ shifting from 3.04 mM in the WT to 5.13 mM in the variant (Fig. 8, Fig. S15, and Table S1).

## Discussion

In this study, we demonstrate that an AI-guided predictive pipeline leveraging LLM-derived ecological priors is an efficient strategy for targeting distal residues to enhance enzymatic activity. To evaluate the systematic predictive power of this approach, we compared it against a preliminary baseline model, which was trained on conventional functional taxonomy rather than ecological metadata. Notably, the baseline model failed to identify any active distal variants among 7 tested candidates (0/7 success rate, Fig. S3), illustrating the limitations of relying solely on broad functional categories. In contrast, our refined framework identified the V129G mutation in SaMccB from a focused search space of only 4 candidates (1/4 success rate), exhibiting an approximate 2-fold increase in catalytic turnover. This divergence suggests that our findings are driven by systematic predictive precision rather than stochastic variation. Although cross-study comparisons should be interpreted cautiously, the number of experimentally tested candidates in this study was considerably smaller than in many directed-evolution workflows [5–7,17,65,78]. Collectively, these results suggest that ecological-label-driven AI predictions can reduce the experimental burden of sequence screening, while our structural and biochemical analyses of V129G support a dynamics-linked gain of function [13,30,79–81].

The model’s performance likely reflects a combination of informative GPT-4o-derived labels and residue-level contextualization by self-attention. Permutation experiments, in which the GPT-4o labels were randomly shuffled, resulted in a marked collapse of model performance, supporting the view that these putative annotations by the LLM GPT-4o provide useful biological constraints for training (Fig. S4). Furthermore, architectural ablation experiments showed that eliminating the self-attention layers substantially reduced model performance (Fig. 3 and Fig. S8). This structural dependency supports the conclusion that the self-attention mechanism contributes to contextualizing the GPT-4o-generated data, effectively mapping the complex functional relationships defined by the putative categories (Fig. 3 and Fig. S8).

Mechanistic relevance is supported by structural and kinetic analysis of the top-ranked variant. The kinetic and structural profile of the V129G variant highlights a classic *K*_M_–*k*_cat_ trade-off, where distal structural softening enhances catalytic turnover at the expense of ground-state substrate binding affinity [30,82,83]. By destabilizing the local hydrophobic packing of the Gly129-containing helix, the mutation relaxes the rigidity of the proximal active site loop and increases the number of available conformers (Fig. 6 and Fig. S13) [84]. This increased plasticity is consistent with lowering the activation energy for rate-limiting conformational transitions, such as product release, which could contribute to the observed increase in *k*_cat_ [79,85–88]. However, this entropy-enhanced turnover speed sacrifices enthalpy-driven substrate stabilization, which explains the concomitant increase in *K*_M_ compared to the rigid, tightly binding WT enzyme. Ultimately, this entropy-driven shift in the catalytic landscape suggests that tuning distal backbone dynamics can modulate the kinetic behavior of this PLP-dependent enzyme.

The connection between flexibility and function is further substantiated by the temperature-dependent activity profile. While V129G outperformed the WT enzyme at physiological temperatures (up to 40 °C), this advantage vanished at 45 °C (Fig. 7). This behavior is consistent with the classical stability–activity trade-off hypothesis [30,89]. The introduced flexibility lowers the energy barrier for catalysis at moderate temperatures but renders the enzyme more susceptible to thermal unfolding or excessive dynamics at elevated temperatures. Thus, V129G appears to improve catalytic throughput within a mesophilic temperature range while preserving the global fold (Fig. 6A). Ultimately, the successful characterization of this distal gain-of-function mutation provides mechanistic support for the utility of our AI-guided approach and suggests that it can identify nonobvious residues relevant to function.

## Conclusion

Our results suggest that our custom-curated AI pipeline, which integrates LLM-based putative annotations and Transformer classification, serves as a proof of concept to reduce the screening burden in family-specialized enzyme engineering. By experimentally screening a few model-prioritized candidates, we identified a highly nonobvious distal mutation that doubled the catalytic turnover. These results suggest that tailoring domain-aware models to a homologous enzyme family may improve prioritization efficiency and interpretability. Moving forward, this modular framework can provide a useful starting point for future studies in other enzyme families.

## Data Availability

The source code supporting this study is available at: https://github.com/iungyu-snu/EnzFormer. The datasets supporting this study are available via Zenodo: doi:10.5281/zenodo.17291636. The coordinates and structure factors for the SaMccB V129G variant have been deposited in the Protein Data Bank under accession code 9XLU.
