# Supplementary material for: Family-Specialized Transformer for L-cystathionine gamma-lyase Engineering and Its Structural Interpretation
Source: Comput Struct Biotechnol J. 2026 Jun 5;35(1):0073. doi: 10.34133/csbj.0073 (PMC13237489; doi:10.34133/csbj.0073)
Supplement: Supplementary 1 — Figs. S1 to 15 Tables S1 to S8 [file csbj.0073.f1.zip › Fig_second_revision_S2.pdf]

A

System Prompt

```
DEFAULT_SYSTEM_PROMPT = """You are labeling a protein dataset for cystathionine gamma-lyase (CGL, EC 4.4.1.1).

Your job is to generate a putative activity label for each natural CGL homolog using only the species of the organism carrying the
    enzyme, as given by the UniProt species name.

Label definitions:
- high: there is evidence of strong selective pressure or a specialized CGL role beyond routine housekeeping.
- low: there is no evidence of a specialized CGL role; the enzyme is presumed to function as a standard housekeeping enzyme.

Interpretation rubric to follow:
- high:
  strong selective pressure
  CGL appears to play a specialized role beyond housekeeping
  examples include H2S production in pathogens or dedicated L-cysteine supply
- low:
  no evidence of specialized function
  presumed standard housekeeping enzyme

Temperature-context rubric:
- thermo: thermophilic context (> 45 C)
- meso: mesophilic context (15-45 C)
- psychro: psychrophilic context (<= 15 C)

Return only schema-conforming JSON with the class labels."""
```

B

Label Schema

```
LABEL_SCHEMA: dict[str, Any] = {
    "type": "object",
    "properties": {
        "activity_label": {
            "type": "string",
            "enum": ["high", "low"],
        },
        "temperature_label": {
            "type": "string",
            "enum": ["thermo", "meso", "psychro"],
        }
    },
    "required": [
        "activity_label",
        "temperature_label",
    ],
    "additionalProperties": False,
}
```
