# Supplementary material for: Family-Specialized Transformer for L-cystathionine gamma-lyase Engineering and Its Structural Interpretation
Source: Comput Struct Biotechnol J. 2026 Jun 5;35(1):0073. doi: 10.34133/csbj.0073 (PMC13237489; doi:10.34133/csbj.0073)
Supplement: Supplementary 1 — Figs. S1 to 15 Tables S1 to S8 [file csbj.0073.f1.zip › Supplementary_figures_tables_with_legends_second_revised.docx]

**SUPPLEMENTAL INFORMATION**

**Figure S1. Dataset curation and class composition.**(a) Number of CGL-related UniProt entries retained after each curation step: initial retrieval (Init), protein-existence filtering (PE filter), per-file deduplication (File dedup), cross-file deduplication (Cross dedup), temperature filtering (Temp filt), and addition of the in silico “No activity” set (No-act added). (b) Final class counts in the three-class dataset: putative High activity, putative Low activity, and No activity

**Figure S2. LLM system prompt and JSON output schema for automated CGL labeling**
(A) System prompt containing instructions and interpretation rubrics for labeling CGL homologs based on species of origin. Activity levels are categorized as high or low based on evidence of selective pressure or specialized physiological roles. Temperature-context labels are defined for thermophilic (> 45 °C), mesophilic (15-45 °C), and psychrophilic (≤ 15 °C) environments.
(B) Structured JSON schema defining the required output format for model responses. The schema specifies activity_label and temperature_label as required properties and enforces consistent classification through predefined enumeration values.

**Figure S3. Experimental validation of loss-of-function anchor variants.** Relative enzymatic activities of selected variants—including F222P, Y26S, and a panel of R249 mutations (F, I, V, and Y)—normalized to the activity of wild-type (WT) CGL. All tested variants consistently exhibited near-baseline relative activity, validating their selection as non-functional anchors for training and benchmarking. The L231P variant was also retained as a non-functional anchor but is not shown due to insufficient protein yields for activity measurement.

**Figure S4. Permutation-based stress test for GPT-4o-derived labels.**Null distributions of classifier performance under 500 label permutations performed under three constraints: global random shuffling, sequence length–binned shuffling, and joint length×amino-acid-composition (PC1)–binned shuffling. Dashed vertical lines indicate observed performance using the original GPT-4o-derived labels. (a) Random Forest Macro-F1. (b) Random Forest MCC. (c) XGBoost Macro-F1. (d) XGBoost MCC.

**Figure S5. Pairwise sequence similarity shows minimal association with GPT-derived activity labels.**Pairwise sequence-similarity analysis was performed using the putative ‘High activity’ and ‘Low activity’ sequences in the dataset, excluding the No_activity class. A total of 20,000 unique unordered sequence pairs were sampled uniformly without replacement, and exact global sequence identity was calculated for each pair using global pairwise alignment.
(A) Distribution of exact global sequence identity for high-high, low-low, and high-low pairs.
(B) Distribution of exact global sequence identity for pairs sharing the same GPT-derived label (same) or different GPT-derived labels (different).
(C) Null distribution of the area under the receiver-operating characteristic curve (AUC) obtained after randomly shuffling sequence labels across the sampled sequences while keeping the precomputed sequence-similarity values fixed. The dashed line indicates the chance-level value (AUC = 0.5), and the red line indicates the observed AUC from the unshuffled data.

**Figure S6. Training metrics across model architectures.**
Evolution of training loss, macro-F1, and Matthews correlation coefficient (MCC) over training epochs for the evaluated configurations. Panels represent: (a) ESMC with linear head, (b) ESMC with MLP head, (c) 0 transformer block, (d) EnzFormer with 1 block, (e) EnzFormer with 3 blocks, (f) EnzFormer with 5 blocks, (g) EnzFormer with FFN-only architecture, and (h) EnzFormer with sequence-level embedding.

**Figure S7. Confusion matrices for architectural ablation study.**
Row-normalized confusion matrices showing classification performance across evaluated model architectures. The axes represent the True and Predicted classes: high activity, low activity, No activity, and reject. The color scale indicates the row-normalized ratio. Panels represent: (a) ESMC with linear head, (b) ESMC with MLP head, (c) 0 transformer block, (d) EnzFormer with 1 block, (e) EnzFormer with 3 blocks, (f) EnzFormer with 5 blocks, (g) EnzFormer with FFN-only architecture, and (h) EnzFormer with sequence-level embedding.

**Figure S8. EnzFormer architectural ablation study.**
Model performance across ablated architectures evaluated on the three-class dataset. Conditions include Transformer depth (Blocks = 0, 1, 3, or 5), attention removal (FFN-only), and pre-pooled sequence-level inputs (Seq-Level). Metrics shown are (top left) Accuracy, (top right) Balanced Accuracy, (bottom left) Macro-F1, and (bottom right) MCC. Bars denote mean performance across runs, with points indicating individual runs.

**Figure S9. Comparative analysis of performance of the EnzFormer on *S. cerevisiae* CGL activity datasets**To prevent data leakage, the EnzFormer was retrained on a dataset from which all sequences sharing more than 80% identity with the *S. cerevisiae* CGL were strictly excluded. (a) Spearman rank correlation coefficients (ρ) and (b) Kendall rank correlation coefficients (τ) between predicted and experimental activity values for EnzFormer and selected baseline models, including ESM-2, PSSM, CataPro, UniKP, and DLKcat.

**Figure S10. Robustness of model performance and generalization under varying sequence identity constraints.**

To evaluate the generalization capability of EnzFormer, the model was retrained on datasets partitioned using sequence-identity-controlled splits at 70% (Seq ID 0.7) and 40% (Seq ID 0.4) thresholds, in addition to standard random partitioning. The performance was assessed using Macro-F1 score, balanced accuracy, and Matthews correlation coefficient (MCC). The consistent performance across these retraining regimes demonstrates EnzFormer’s robustness in predicting activities for sequences with low similarity to the training set.

**Figure S11. Random-split, species-holdout, and genus-holdout performance of EnzFormer.**

EnzFormer performance under random 5-fold, species-grouped 5-fold, and genus-grouped 5-fold evaluation after excluding *No activity* samples. Bars represent the mean across three random seeds (21, 89, and 178), and error bars indicate standard deviation. Metrics shown are Macro-F1, balanced accuracy, MCC, and high-activity F1.

**Figure S12. Effect of ‘No activity’ sample removal on predictive performance**

Comparison of Natural-class average F1 scores between original baseline models and variants retrained after the exclusion of no-activity samples (Natural-only). Comparisons are provided for original, seqid40, and seqid70 dataset configurations. Data represent mean plus-or-minus sample standard deviation from five-fold cross-validation.

**Figure S13. MD-based analysis of local hydrophobic packing around the V129G helix.**
(a) Frame-wise distribution of the total number of hydrophobic side-chain contacts involving helix residues 124-134, pooled across five independent MD trajectories for WT and V129G.

(b) Contact rewiring map showing the change in residue-pair contact occupancy (V129G - WT) between hydrophobic residues in residues 124-134 (y-axis) and hydrophobic partner residues (x-axis). A contact was defined when the closest heavy-atom distance between a residue pair was < 0.45 nm. Contact occupancy was calculated separately for each replicate as the fraction of analyzed frames in which the contact was present, and values were then averaged across five replicates per system. Warm colors indicate increased contacts in V129G, whereas cool colors indicate decreased contacts.

**Figure S14. Representative SDS-PAGE gels showing the time-course digestion of WT and V129G CGL proteins in the absence of PLP.**

Limited proteolysis assay of WT and V129G. Representative SDS-PAGE gel shows limited proteolysis of WT and V129G proteins. CGL and CP1-8 indicate the full-length cystathionine gamma-lyase and its major cleavage products, respectively. The gel shows a time course (0–40 min) of proteolysis in the apo (0 µM PLP).

**Figure S15. AMC fluorescence standard curves for enzymatic activity quantification**
(a,b) AMC fluorescence standard curves measured on the same plates used for the corresponding kinetic assays for WT (a) and V129G (b). Data points represent mean values and error bars indicate standard deviation.

**Table S1. Steady-state kinetic parameters of wild-type and V129G enzymes.**

| **Variant** | **K_m_ (mM)** **(95% CI)** | **Vmax (µM/min)** **(95% CI)** | **R^2^** | **k_cat_ (s^-1^)** **(95% CI)** |
| --- | --- | --- | --- | --- |
| Wild-type | 3.04 (2.78-3.30) | 2.87 (2.77-2.98) | 0.872 | 0.01596 (0.01537-0.01655) |
| V129G | 5.13 (4.19-6.07) | 5.27 (4.59-5.95) | 0.936 | 0.0293 (0.0255-0.0330) |

**Table S2. Sequences of the oligonucleotides used in this study**

**Primer Direction Sequence (5′ → 3′)**

E306Y Forward atcaattgtttggcactatatgtatttttcacttcaaatgcaatcacacctgt

Reverse acaggtgtgattgcatttgaagtgaaaaatacatatagtgccaaacaattgat

H284D Forward gccatatggacatcatcatttaaatgactttcaatacttggatggaa

Reverse ttccatccaagtattgaaagtcatttaaatgatgatgtccatatggc

H284Y Forward gccatatggacatcataatttaaatgactttcaatacttggatggaa

Reverse ttccatccaagtattgaaagtcatttaaattatgatgtccatatggc

V129G Forward ttgggcgtatcgcttgtccaattgaatctgtatgcg

Reverse cgcatacagattcaattggacaagcgatacgcccaa

**Table S3. X-ray Data Collection and Refinement Statistics for the CGL V129G Variant**

| **Item** | **Value** |
| --- | --- |
| Beam line | PLS-II 7A |
| Wavelength (Å) | 0.9793 |
| Space group | I 2 2 2 (No. 23) |
| Cell dimensions (Å; °) | a = 62.832, b = 80.467, c = 161.067; α = β = γ = 90° |
| Resolution (Å) | 40.27–2.33 |
| Rmerge | 0.027 |
| Rpim | 0.027 |
| I/σI | 25.23 |
| Completeness (%) | 87.47 (EDS/DCC) |
| Redundancy | 20 |
| Wilson B-factor (Å²) | 26.28 |
| Refinement (program) | PHENIX |
| Refinement resolution (Å) | 40.27–2.33 |
| No. reflections (work+test) | 15,643 total; test set 1,560 (9.97%) |
| Rwork / Rfree | 0.207 / 0.260 (depositor stats) |
| Total atoms | 2,782 |
| RMS deviations | RMSZ (bond/angle) = 0.16 / 0.34 |
| Ramachandran plot | Favored 97.0%; Allowed 3.0%; Outliers 0.0% |
| PDB code | 9XLU |

**Table S4. Average B-factors of WT and V129G CGL in Selected Structural Regions**

| **Region** | **WT (Å²)** | **V129G (Å²)** | **Δ (V129G − WT) (Å²)** |
| --- | --- | --- | --- |
| overall | 30.66 | 35.72 | +5.06 |
| 95–103 | 42.11 | 61.40 | +19.28 |
| 125–133 | 31.01 | 42.00 | +10.99 |

**Table S5. Remodeling of local hydrophobic contacts in the V129G variant.**

| **Helix residue** | **Partner residue** | **WT occupancy (mean ± SD)** | **V129G occupancy (mean ± SD)** | **Δ occupancy** |
| --- | --- | --- | --- | --- |
| Ile128 | Ile93 | 0.671 ± 0.149 | 0.180 ± 0.145 | -0.492 |
| Ile128 | Val120 | 0.757 ± 0.086 | 0.527 ± 0.395 | -0.230 |
| Ile128 | Leu139 | 0.870 ± 0.145 | 0.652 ± 0.159 | -0.218 |
| Ile128 | Ile141 | 0.122 ± 0.081 | 0.088 ± 0.106 | -0.034 |
| Ile128 | Leu166 | 0.003 ± 0.003 | 0.002 ± 0.003 | -0.001 |
| Ile128 | Ile160 | 0.758 ± 0.084 | 0.889 ± 0.101 | +0.131 |
| Val129 | Ile160 | 0.926 ± 0.022 | 0.672 ± 0.149 | -0.253 |
| Ile132 | Ile93 | 0.909 ± 0.055 | 0.729 ± 0.143 | -0.180 |
| Ile132 | Val120 | 0.004 ± 0.009 | 0.001 ± 0.001 | -0.004 |
| Ile132 | Ala161 | 0.000 ± 0.000 | 0.018 ± 0.029 | +0.018 |
| Ile132 | Leu166 | 0.911 ± 0.021 | 0.972 ± 0.014 | +0.061 |
| Ile132 | Ile160 | 0.680 ± 0.064 | 0.839 ± 0.047 | +0.159 |
| Ile132 | Leu139 | 0.017 ± 0.021 | 0.226 ± 0.217 | +0.209 |

**Table S6. Attribution rank of the substituted site in representative sequences, with WT reference rows for positions 129, 284, and 306.**

| **Sequence** | **Residue evaluated** | **IG rank** | **Attention rank** |
| --- | --- | --- | --- |
| V129G | 129 (G) | 29/380 | 28/380 |
| WT reference | 129 (V) | 366/380 | 177/380 |
| H284Y | 284 (Y) | 14/380 | 13/380 |
| H284D | 284 (D) | 1/380 | 2/380 |
| WT reference | 284 (H) | 18/380 | 78/380 |
| E306Y | 306 (Y) | 7/380 | 15/380 |
| WT reference | 306 (E) | 12/380 | 28/380 |

**Table S7. Recurrently attributed residues across five representative sequences based on integrated gradients and attention.**

| Residue  index | Median  IG rank | IG rank  range | IG top-10  count (n/5) | Median  attention rank | Attention  rank range | Attention top-10  count (n/5) |
| --- | --- | --- | --- | --- | --- | --- |
| 350 | 3 | 3-11 | 4 | 2 | 2-4 | 5 |
| 380 | 1 | 1-5 | 5 | 5 | 2-7 | 5 |
| 304 | 2 | 2-8 | 5 | 10 | 6-13 | 3 |
| 110 | 8 | 7-10 | 5 | 8 | 7-10 | 5 |
| 17 | 6 | 4-10 | 5 | 12 | 12-14 | 0 |
| 35 | 5 | 3-6 | 5 | 14 | 10-15 | 1 |
| 3 | 8 | 5-10 | 5 | 18 | 16-38 | 0 |
| 39 | 11 | 9-18 | 2 | 16 | 15-25 | 0 |
| 41 | 21 | 20-31 | 0 | 9 | 8-14 | 4 |
| 1 | 21 | 16-26 | 0 | 11 | 9-17 | 2 |
| 379 | 28 | 28-37 | 0 | 10 | 7-11 | 3 |
| 10 | 4 | 4-6 | 5 | 37 | 35-41 | 0 |
| 99 | 44 | 32-46 | 0 | 4 | 3-5 | 5 |
| 193 | 76 | 73-84 | 0 | 5 | 3-7 | 5 |
| 318 | 91 | 68-100 | 0 | 6 | 6-11 | 4 |
| 185 | 121 | 116-141 | 0 | 1 | 1-1 | 5 |

*Residues shown were present in the top 10 by integrated gradients or attention in at least two of the five representative sequences (WT, V129G, H284Y, H284D, and E306Y). Ranks were assigned within each sequence, with rank 1 indicating the highest attribution score.*

**Table S8. Sensitivity of EnzFormer-guided candidate prioritization to the ΔPSSM cutoff**

| **ΔPSSM cutoff** | **Filtered pool size** | **E306Y rank** | **H284Y rank** | **V129G rank** | **H284D rank** |
| --- | --- | --- | --- | --- | --- |
| ≥ -9 | 6,559 | 1 | 3 | 5 | 2 |
| ≥ -8 | 5,805 | 1 | 2 | 4 | — |
| ≥ -7 | 4,823 | 1 | 2 | 3 | — |
| ≥ -6 | 3,422 | — | 1 | — | — |
