# Supplementary figures and images for: Family-Specialized Transformer for L-cystathionine gamma-lyase Engineering and Its Structural Interpretation
Source: Comput Struct Biotechnol J. 2026 Jun 5;35(1):0073. doi: 10.34133/csbj.0073 (PMC13237489; doi:10.34133/csbj.0073)

**A**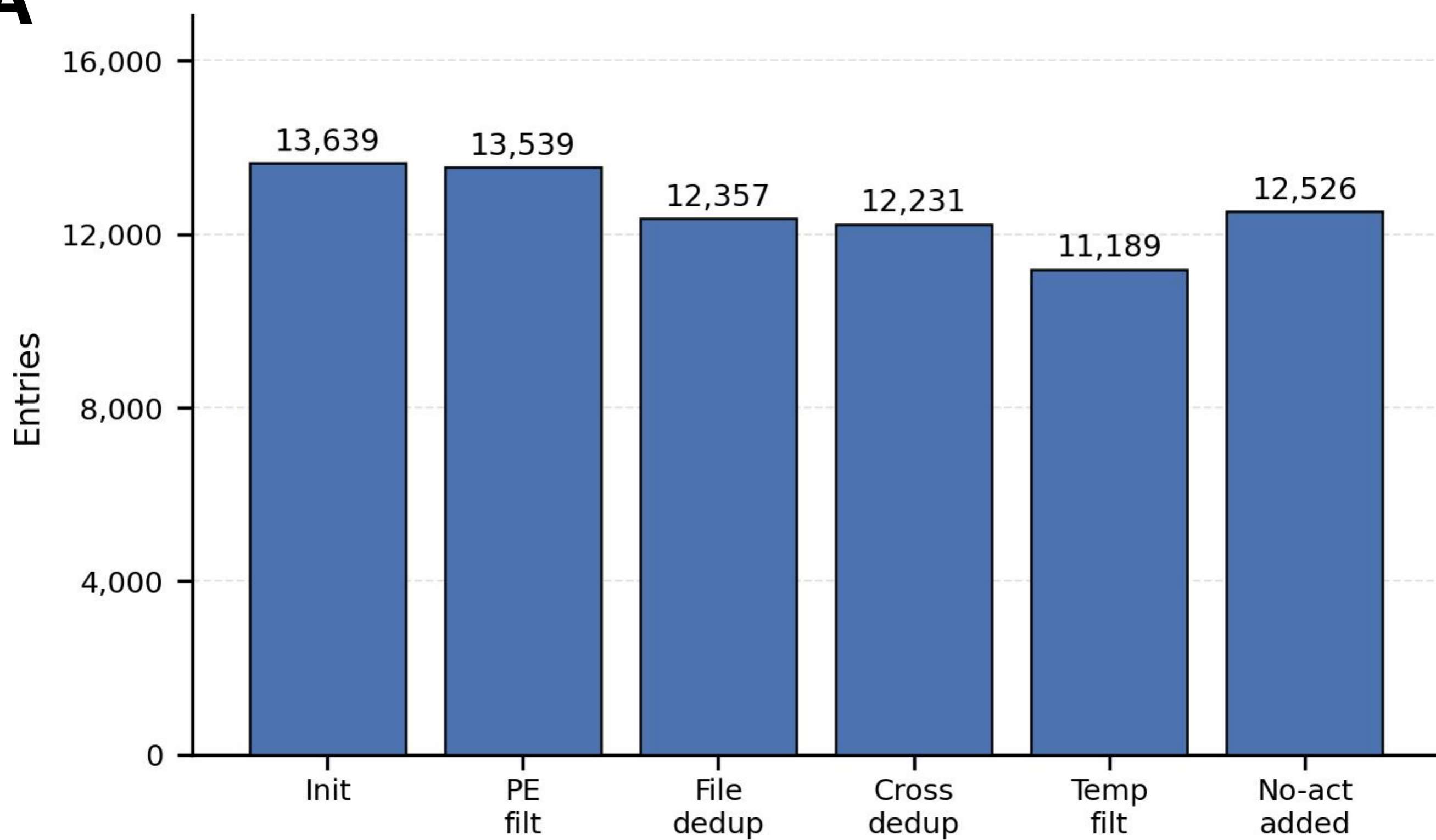**B**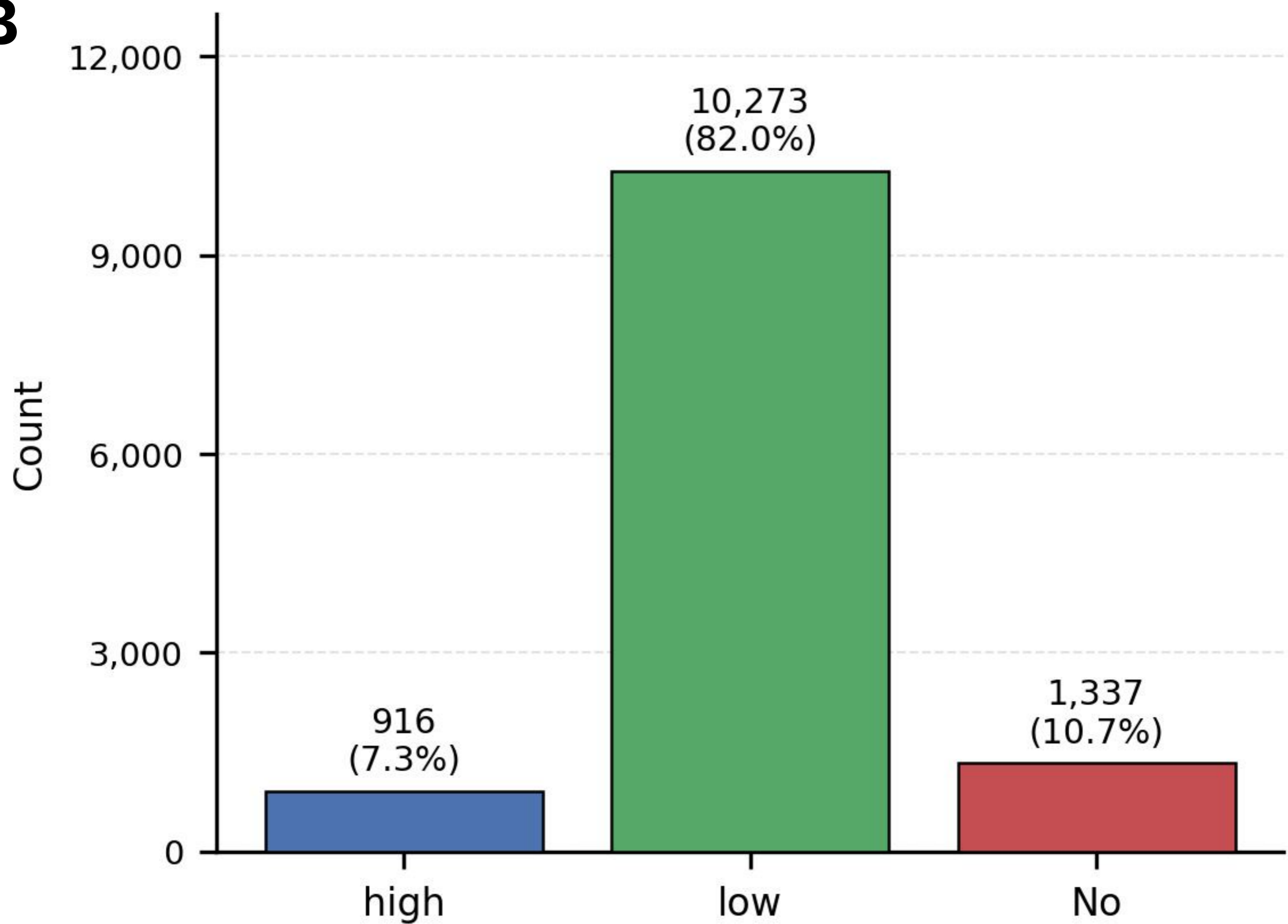

Supplement: Supplementary 1 — Figs. S1 to 15 Tables S1 to S8 [file csbj.0073.f1.zip › Fig_S1.pdf]

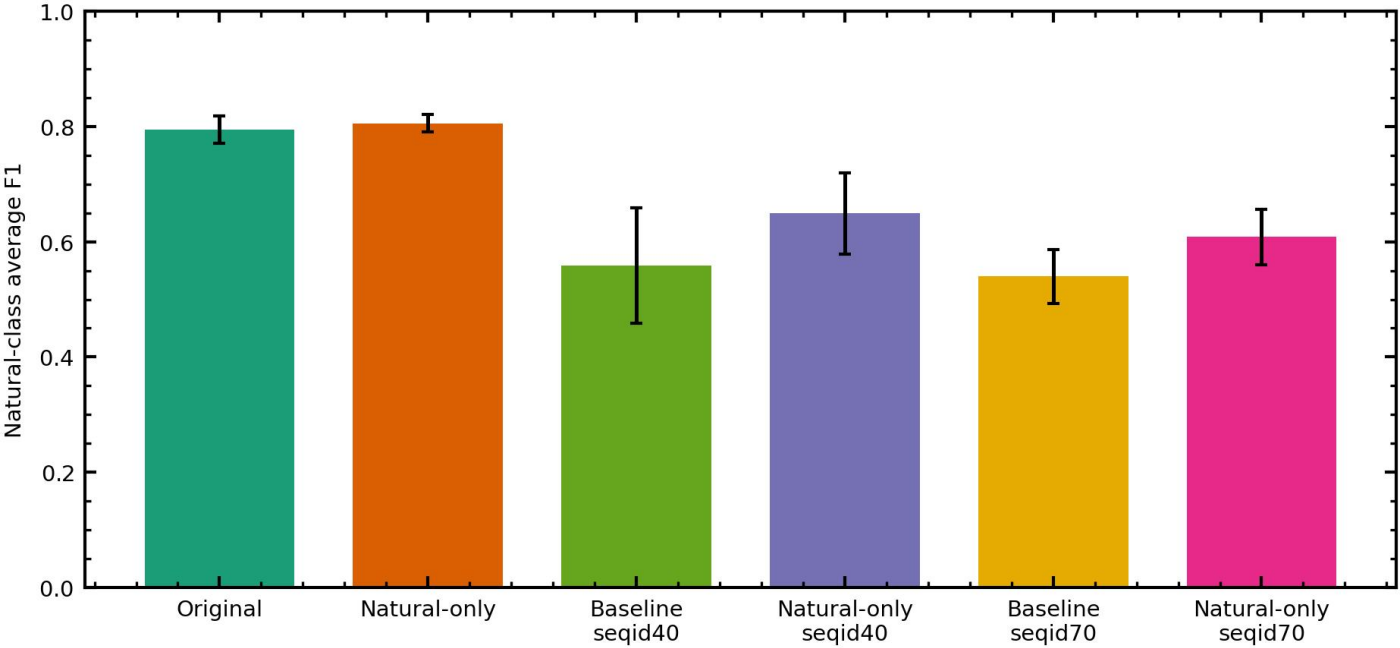

Supplement: Supplementary 1 — Figs. S1 to 15 Tables S1 to S8 [file csbj.0073.f1.zip › Fig_S12.pdf]

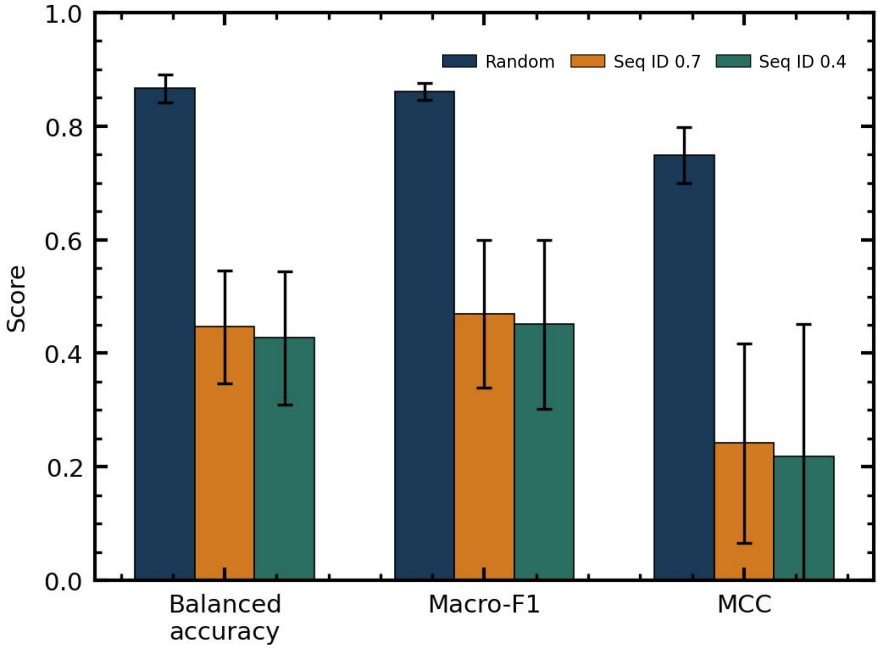

Supplement: Supplementary 1 — Figs. S1 to 15 Tables S1 to S8 [file csbj.0073.f1.zip › Fig_second_revision_S10.pdf]

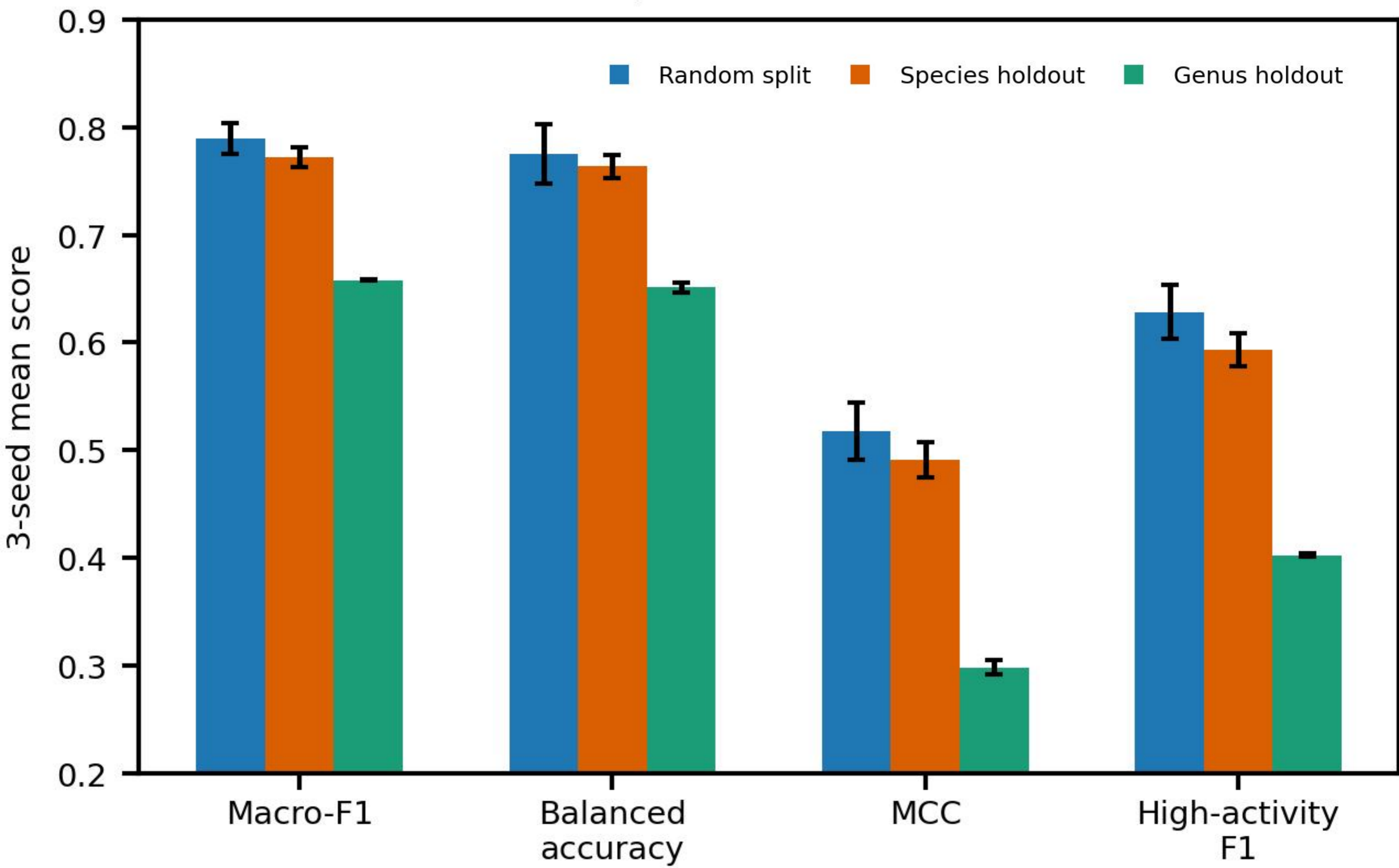

Supplement: Supplementary 1 — Figs. S1 to 15 Tables S1 to S8 [file csbj.0073.f1.zip › Fig_second_revision_S11.pdf]

**A**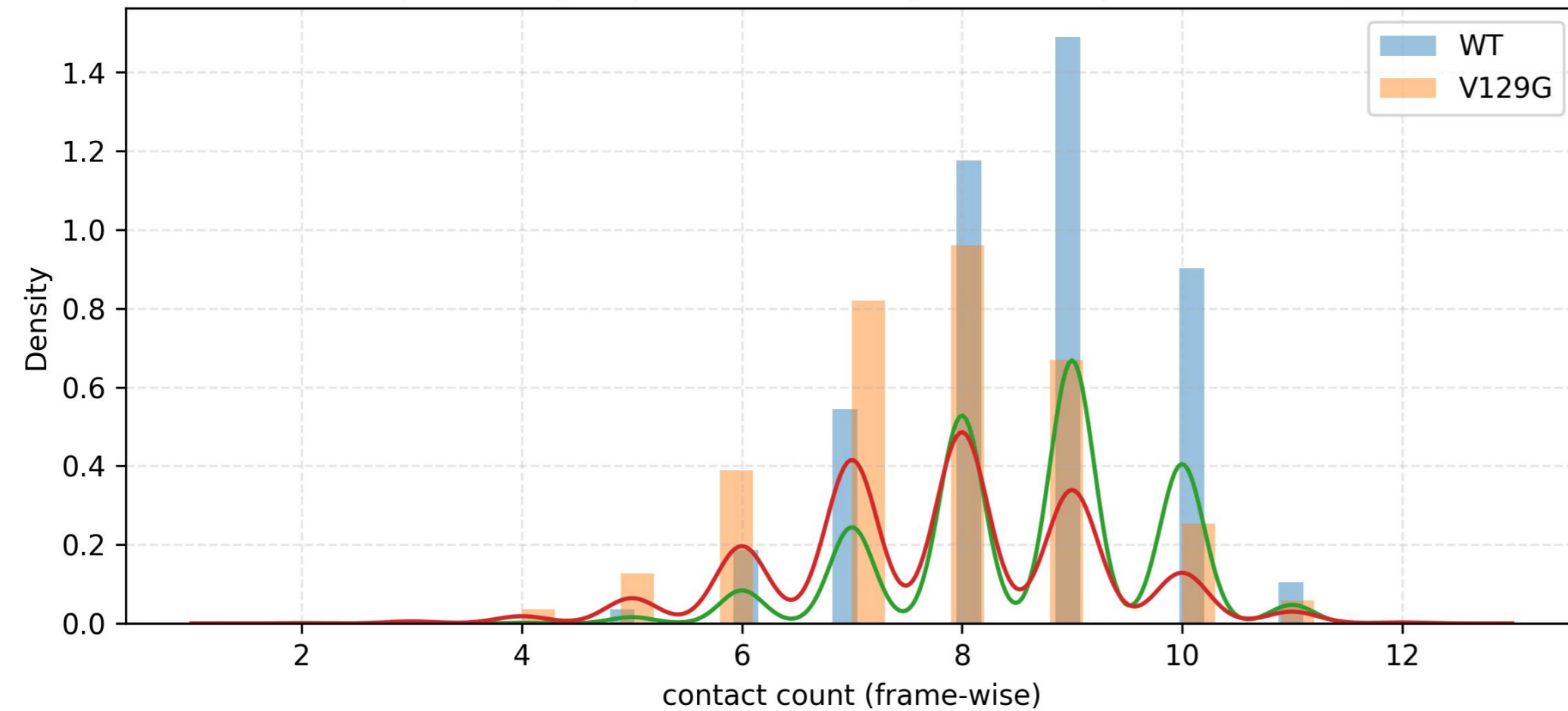**B**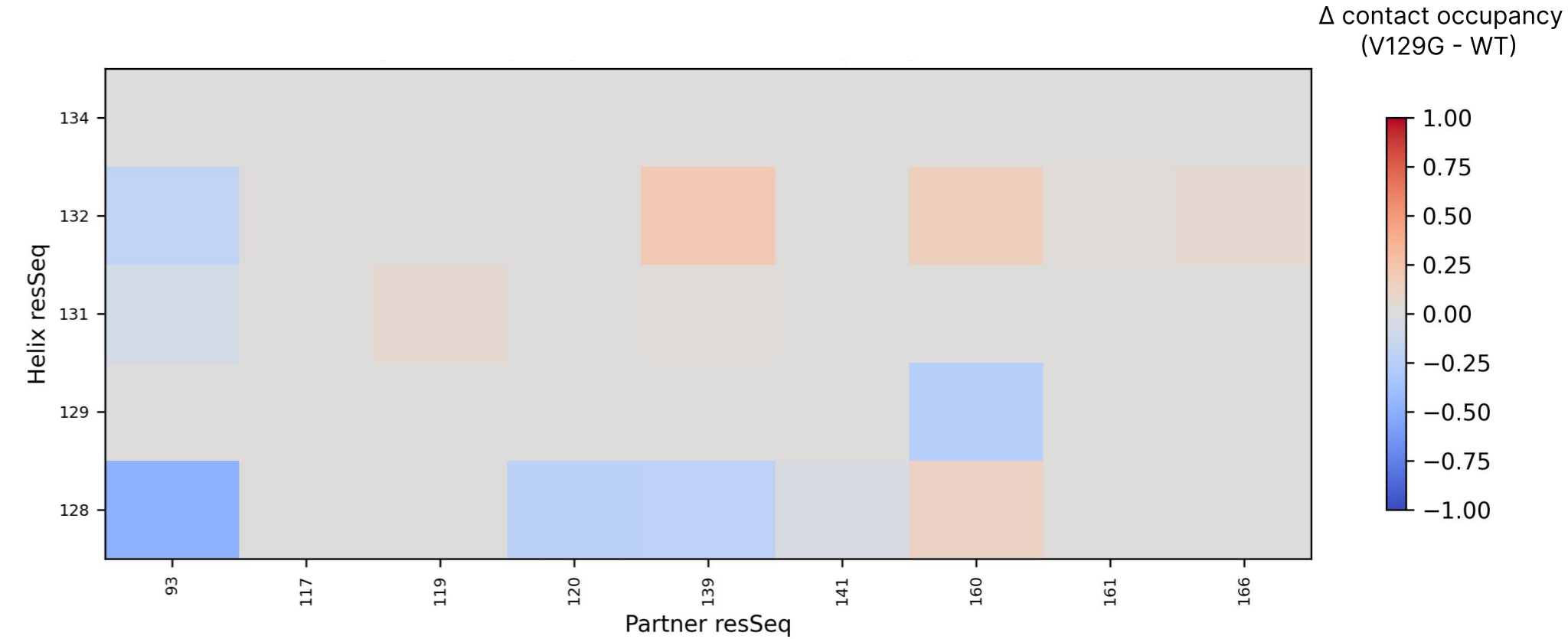

Supplement: Supplementary 1 — Figs. S1 to 15 Tables S1 to S8 [file csbj.0073.f1.zip › Fig_second_revision_S13.pdf]

**WT**

**V129G**

**CGL**

**CP1-8**

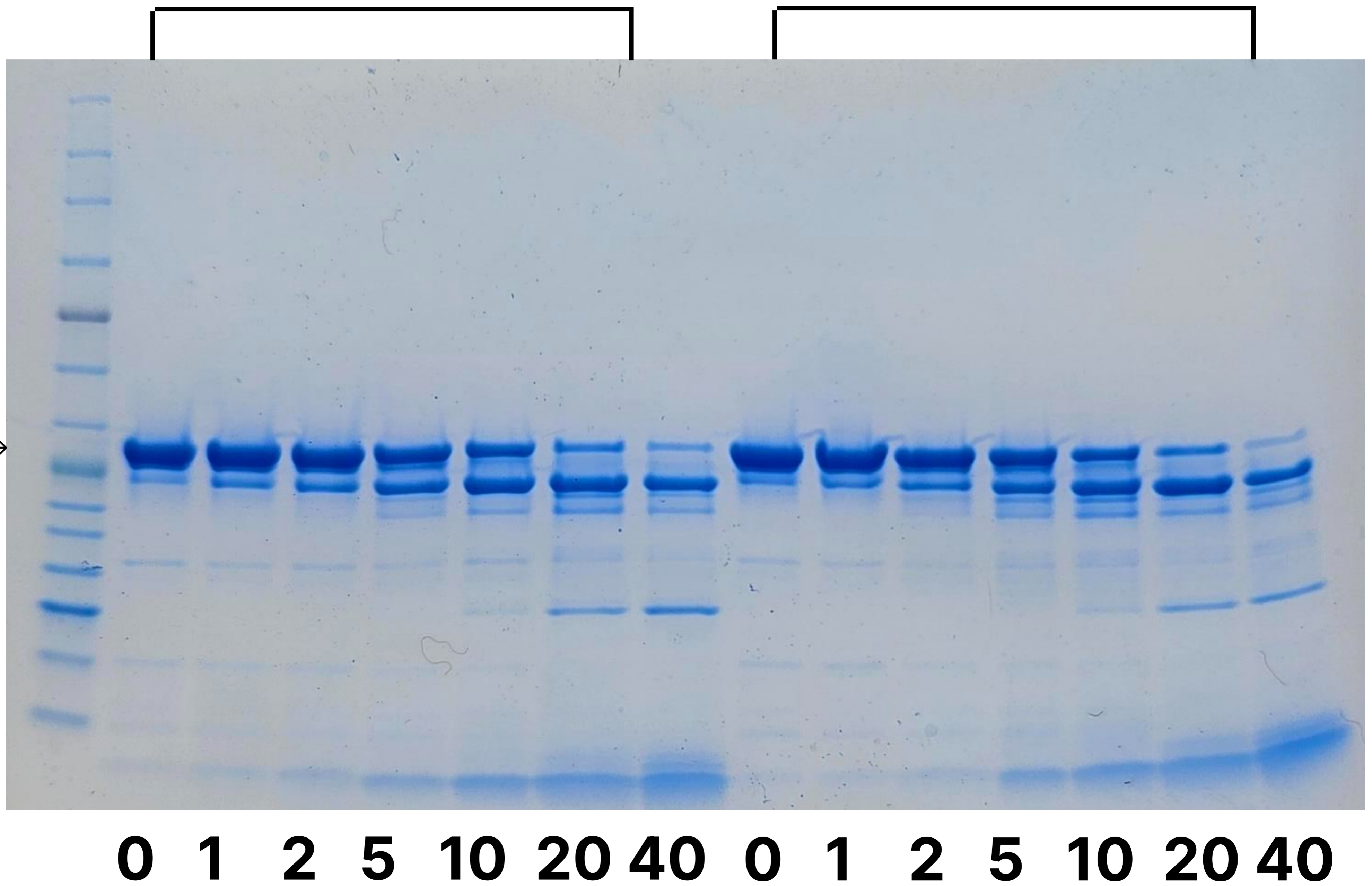

Supplement: Supplementary 1 — Figs. S1 to 15 Tables S1 to S8 [file csbj.0073.f1.zip › Fig_second_revision_S14.pdf]

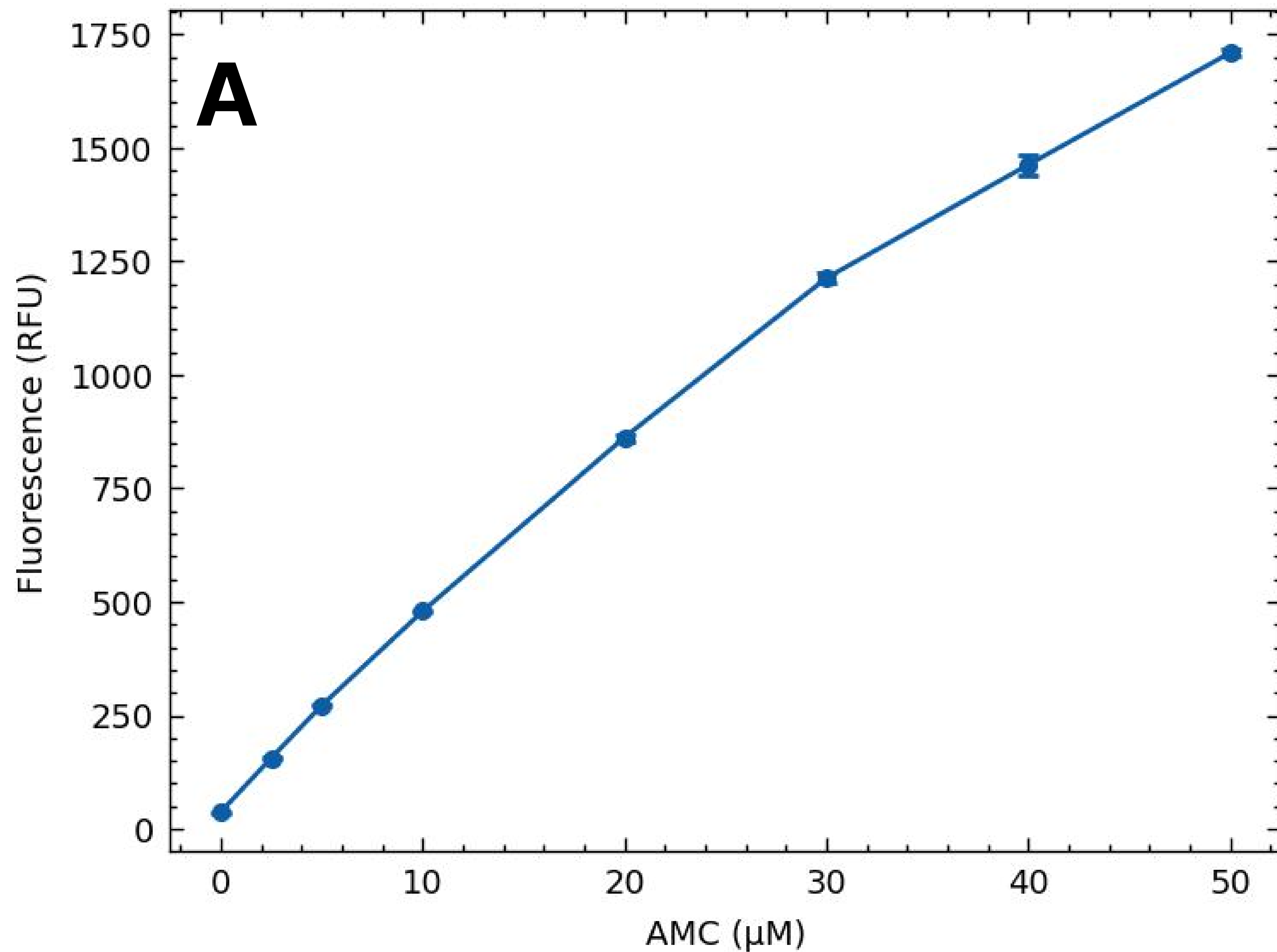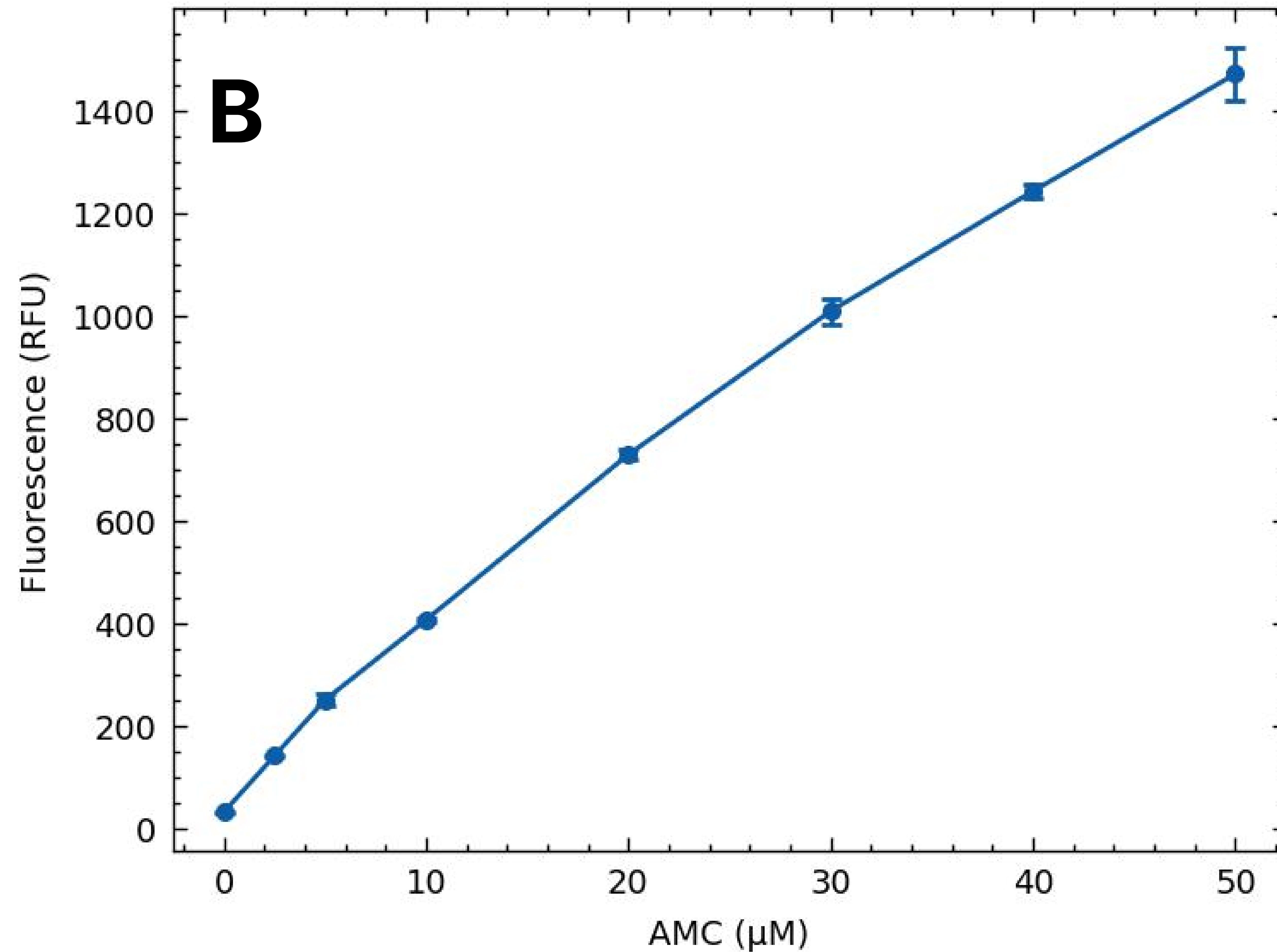

Supplement: Supplementary 1 — Figs. S1 to 15 Tables S1 to S8 [file csbj.0073.f1.zip › Fig_second_revision_S15.pdf]

Relative endpoint activity

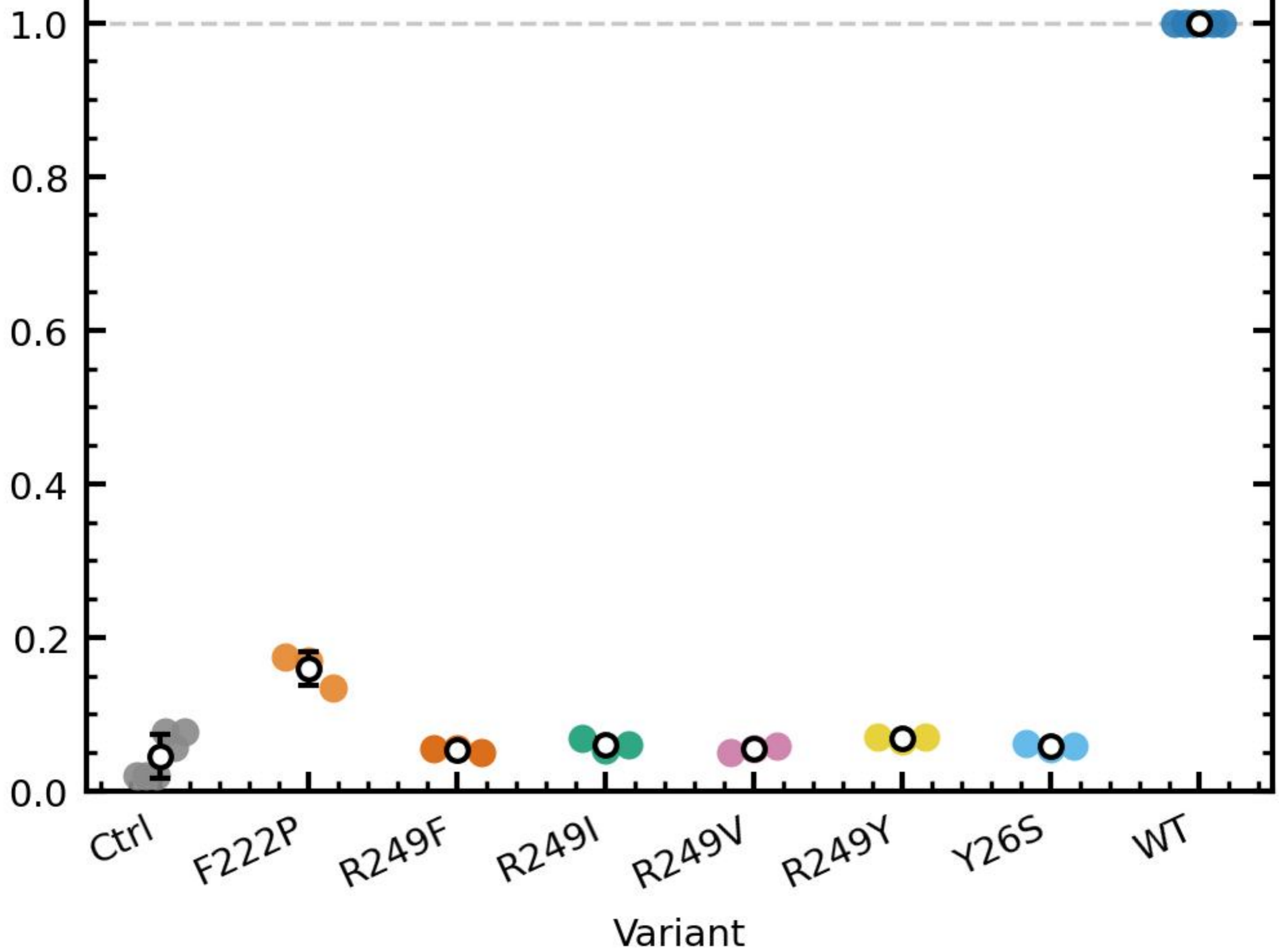

Supplement: Supplementary 1 — Figs. S1 to 15 Tables S1 to S8 [file csbj.0073.f1.zip › Fig_second_revision_S3.pdf]

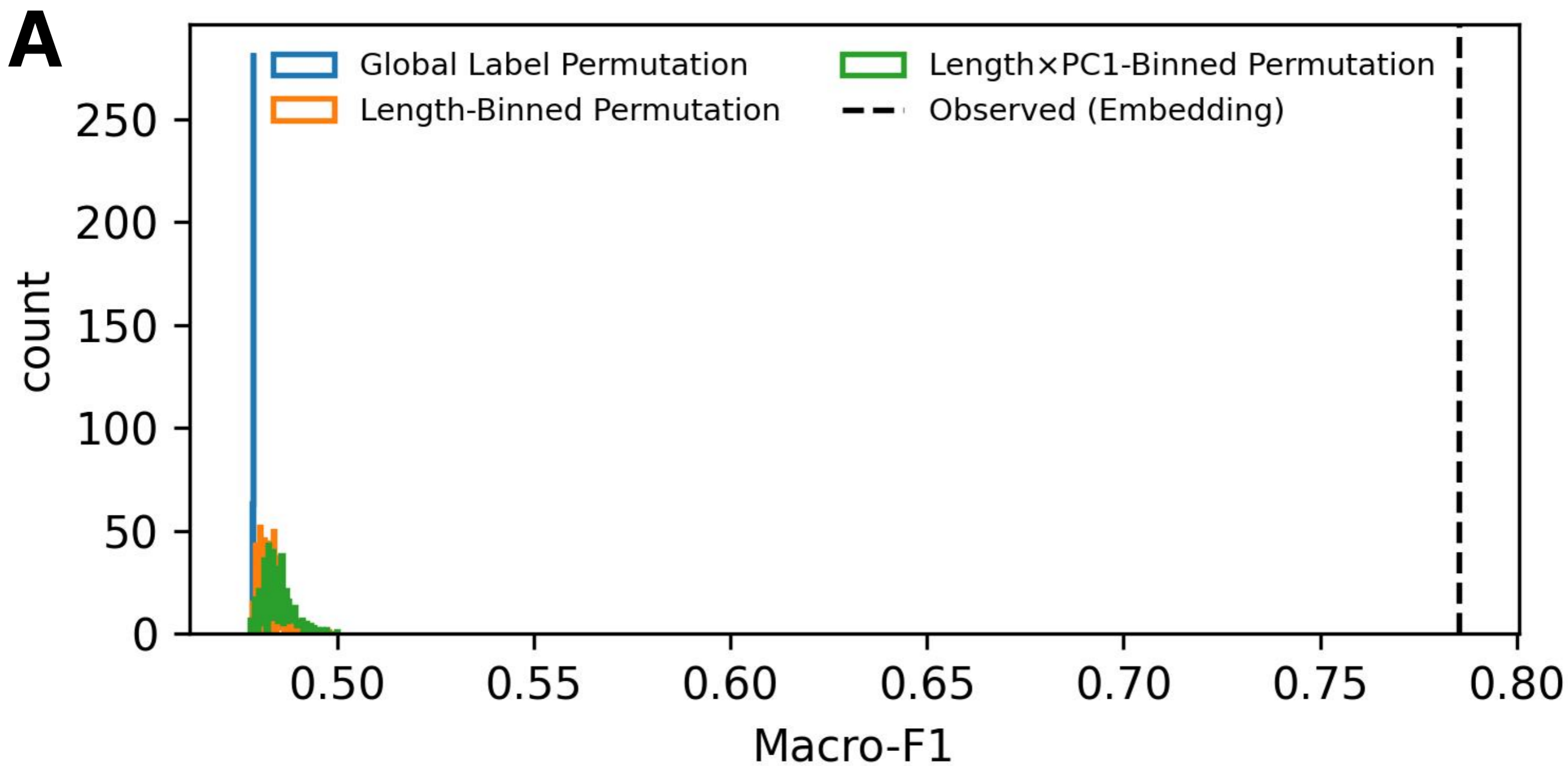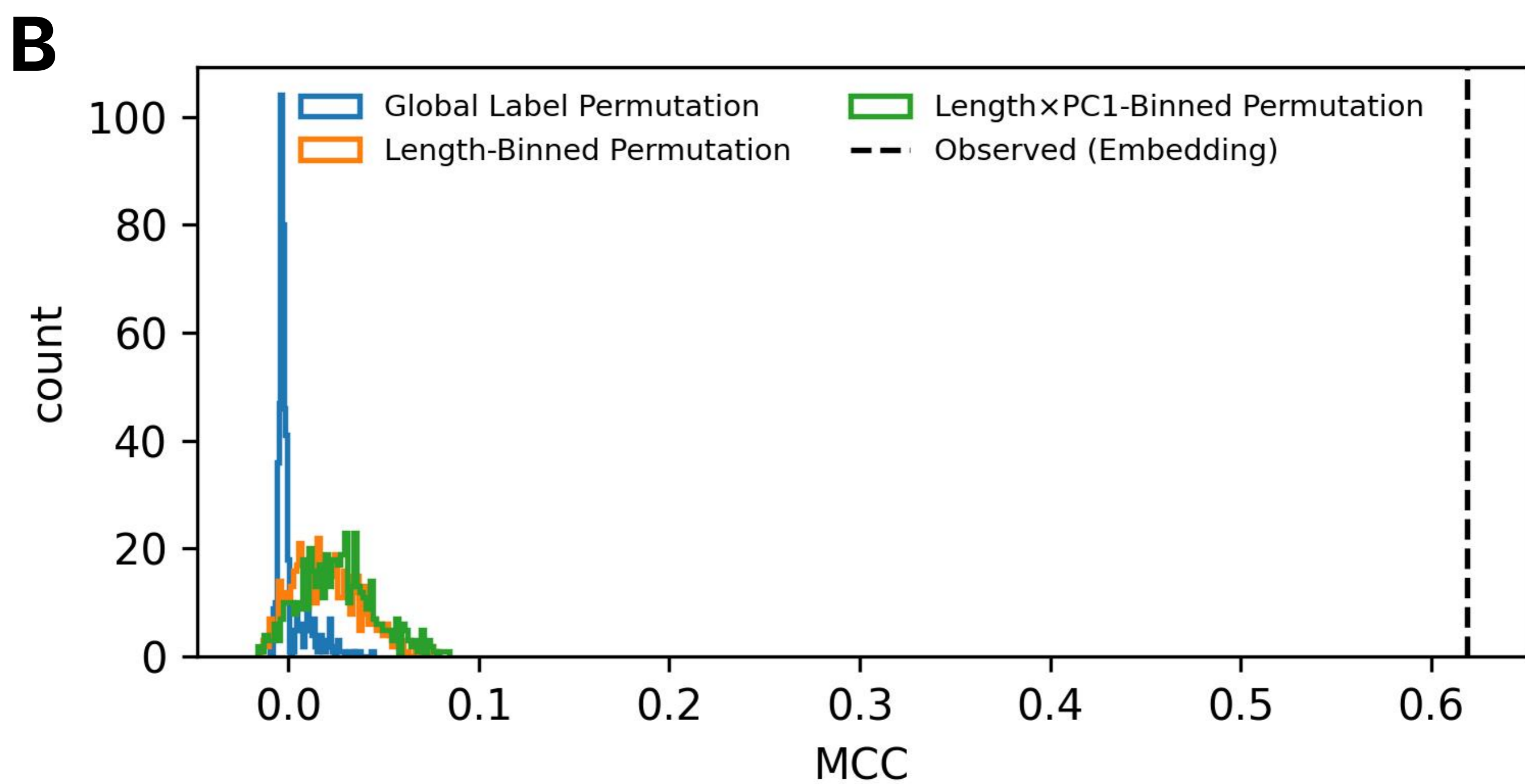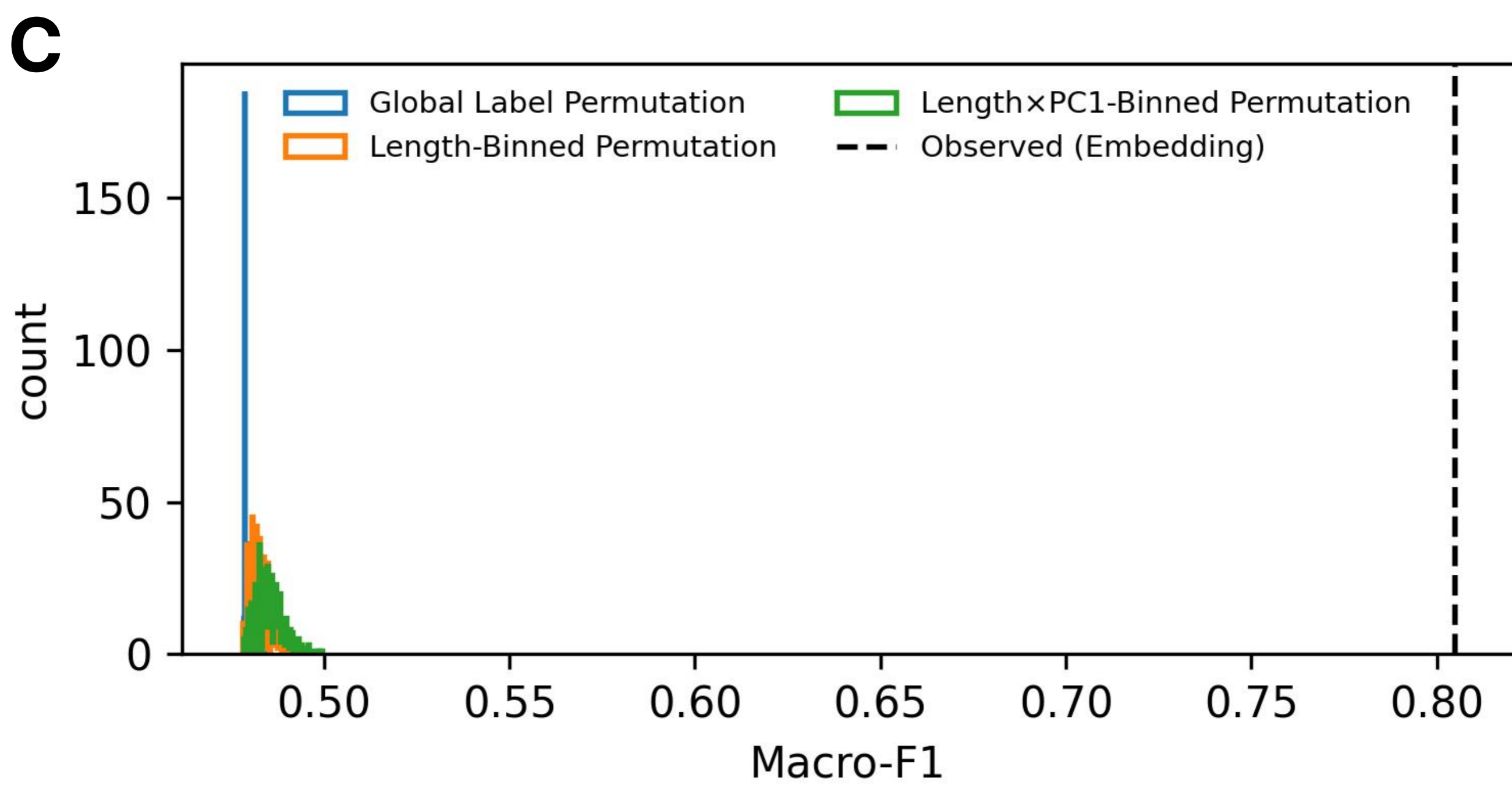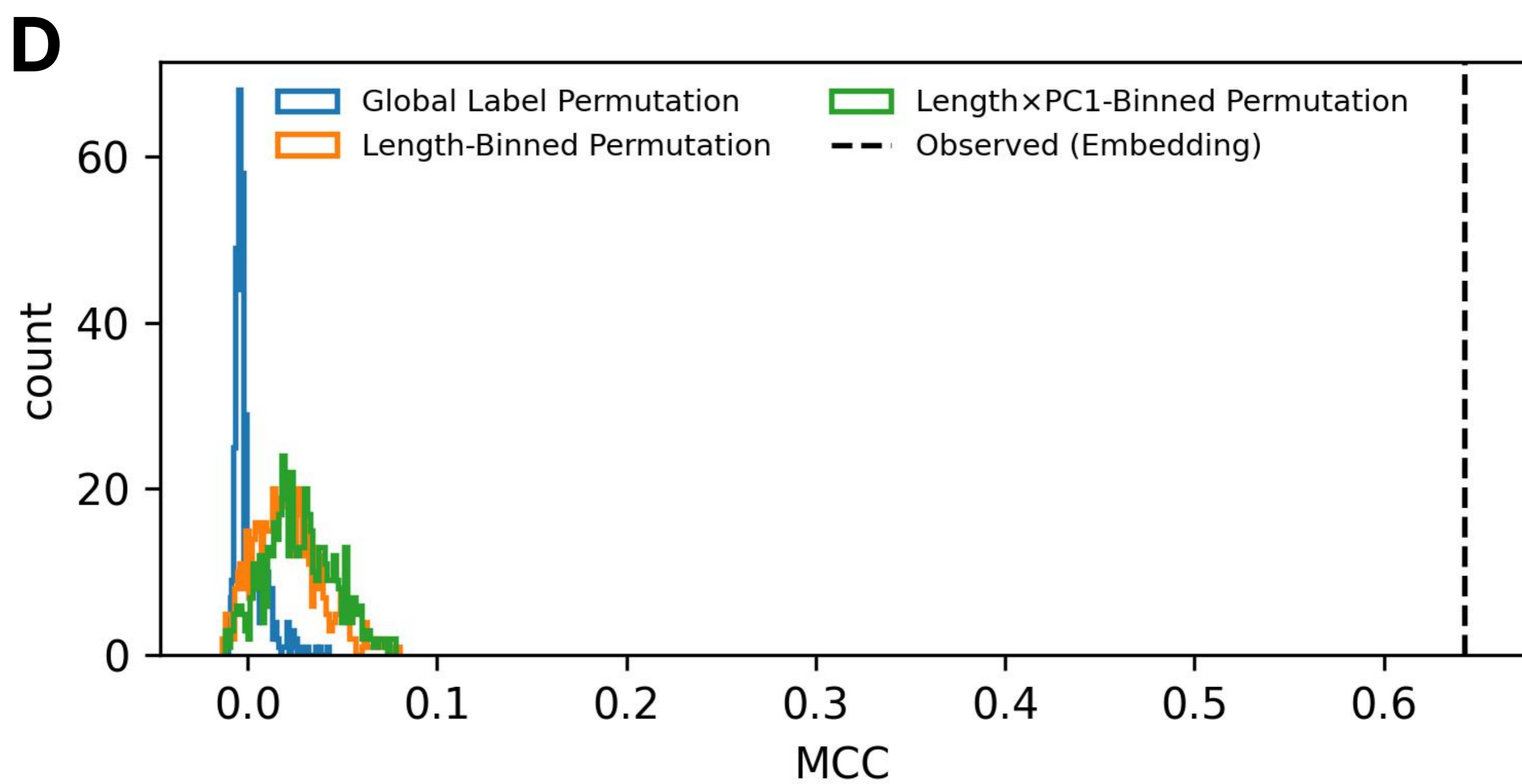

Supplement: Supplementary 1 — Figs. S1 to 15 Tables S1 to S8 [file csbj.0073.f1.zip › Fig_second_revision_S4.pdf]

**A**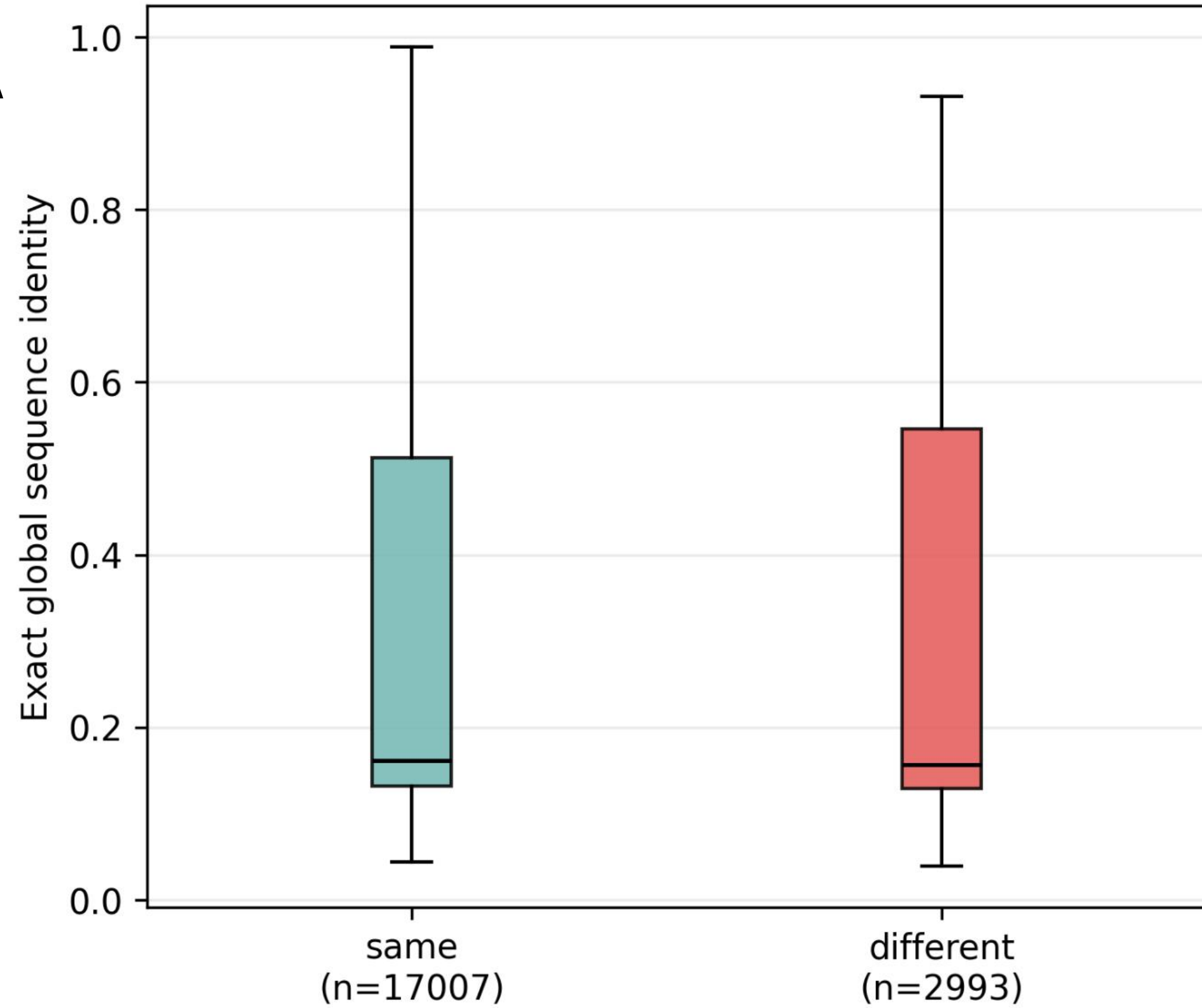**B**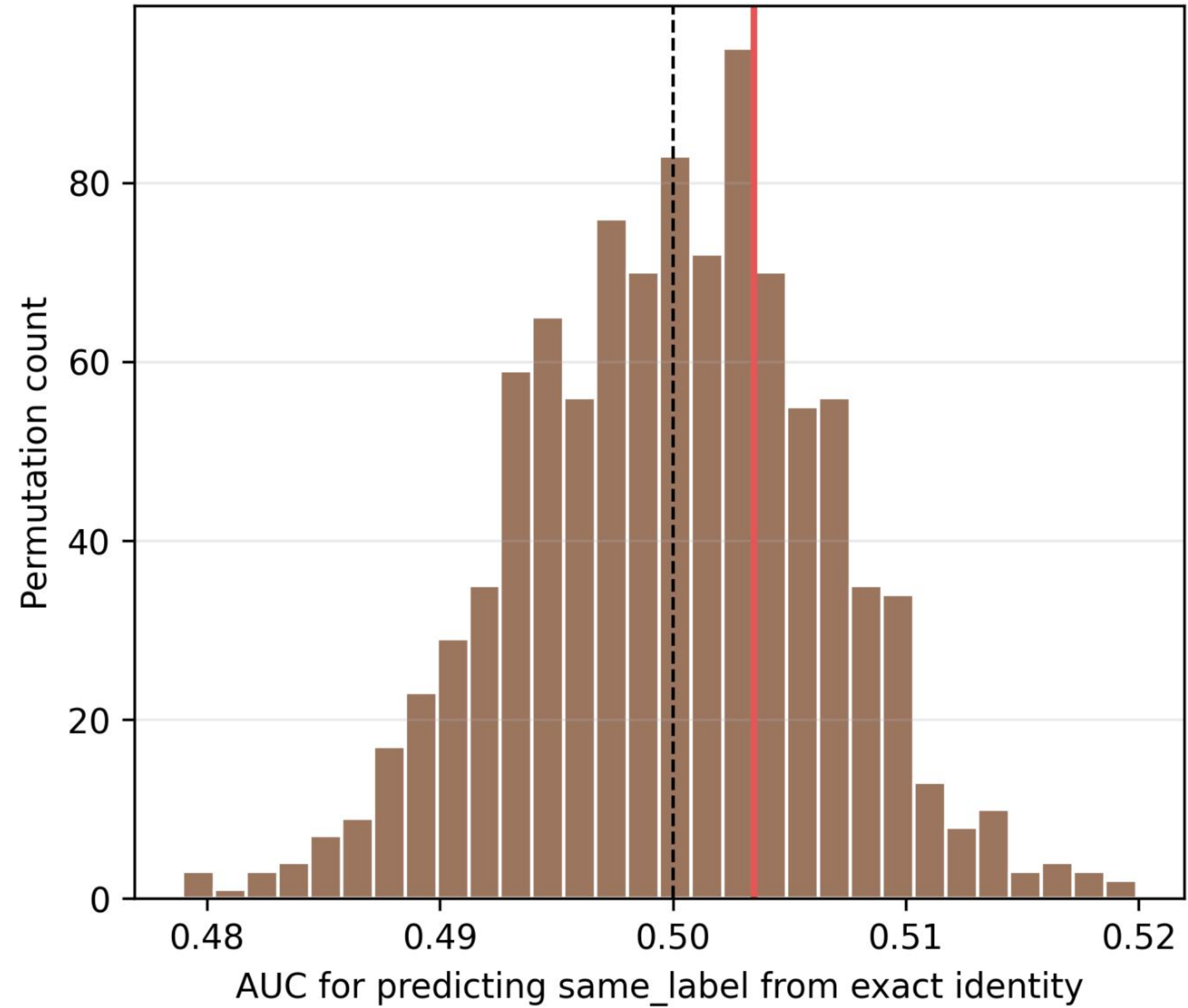

Supplement: Supplementary 1 — Figs. S1 to 15 Tables S1 to S8 [file csbj.0073.f1.zip › Fig_second_revision_S5.pdf]

**A**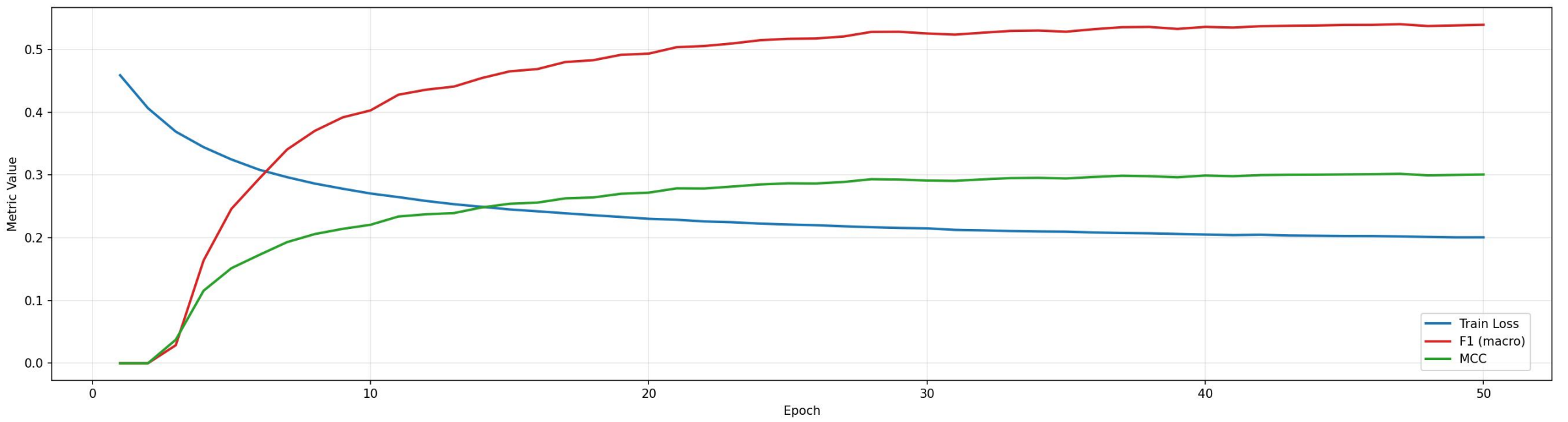**B**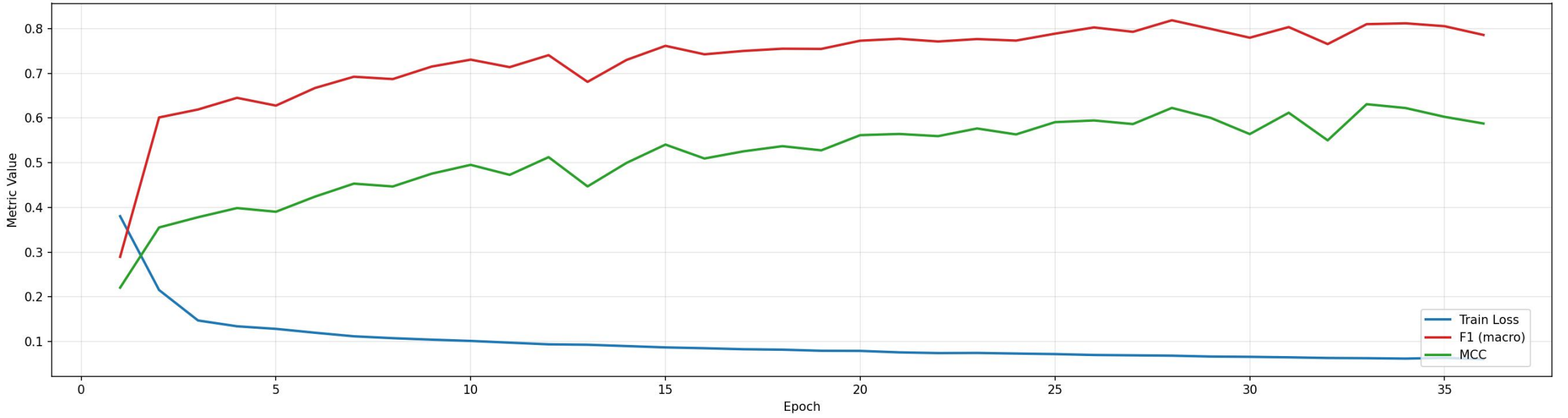**C**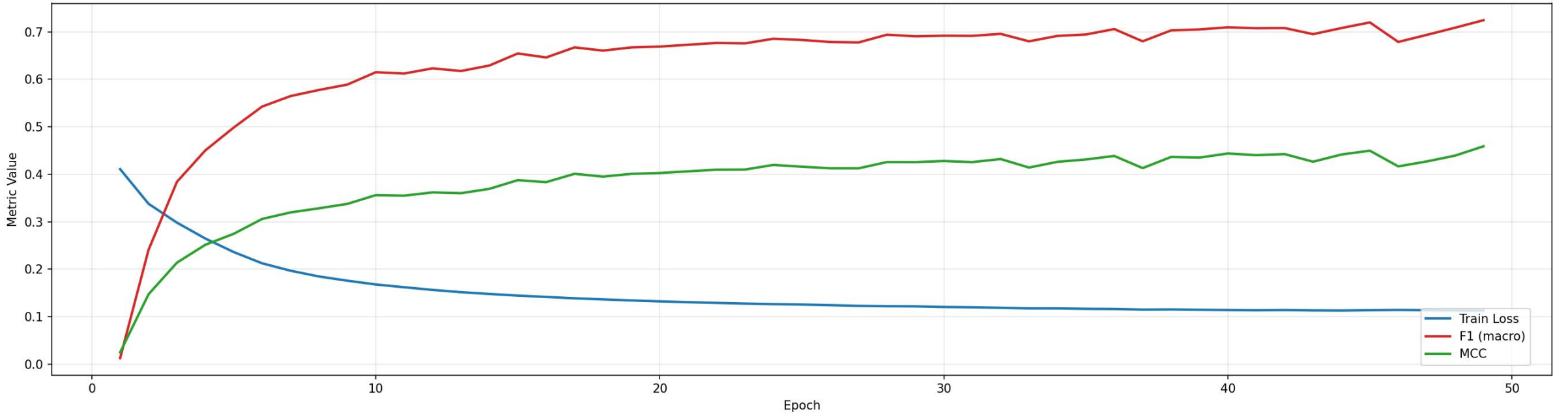**D**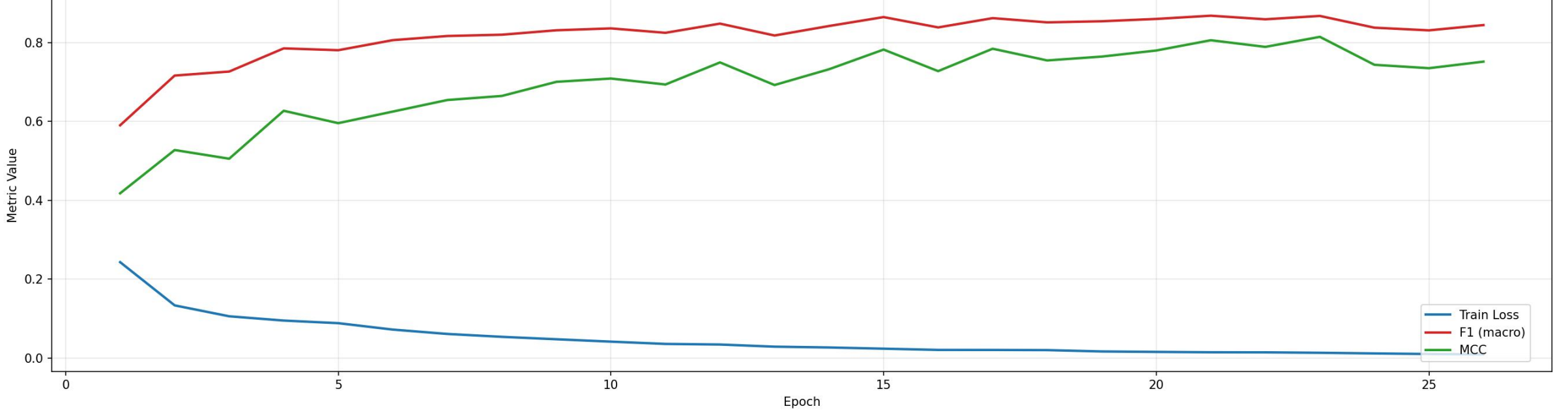**E**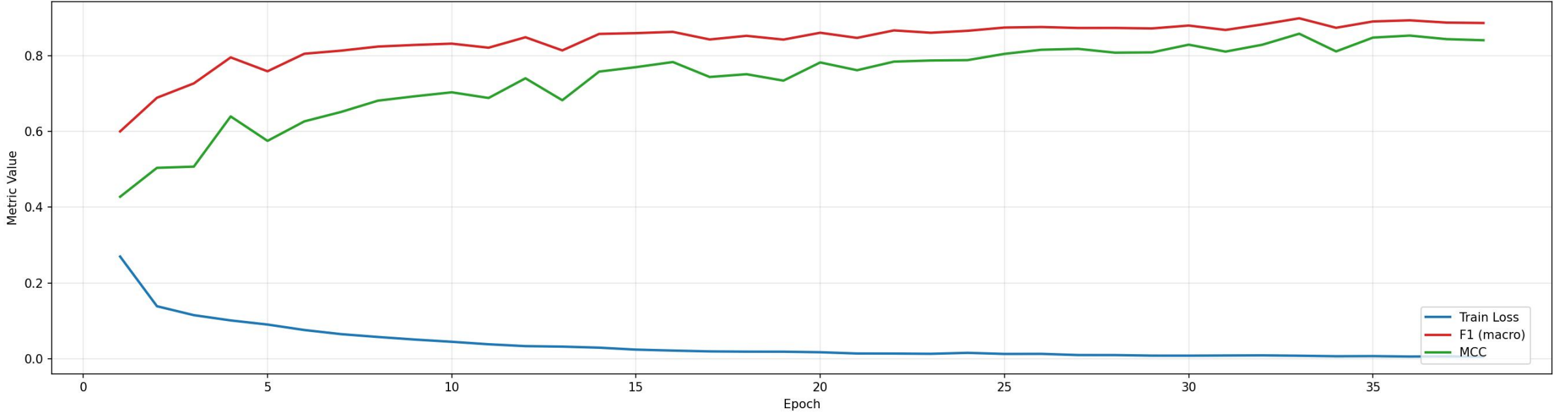**F**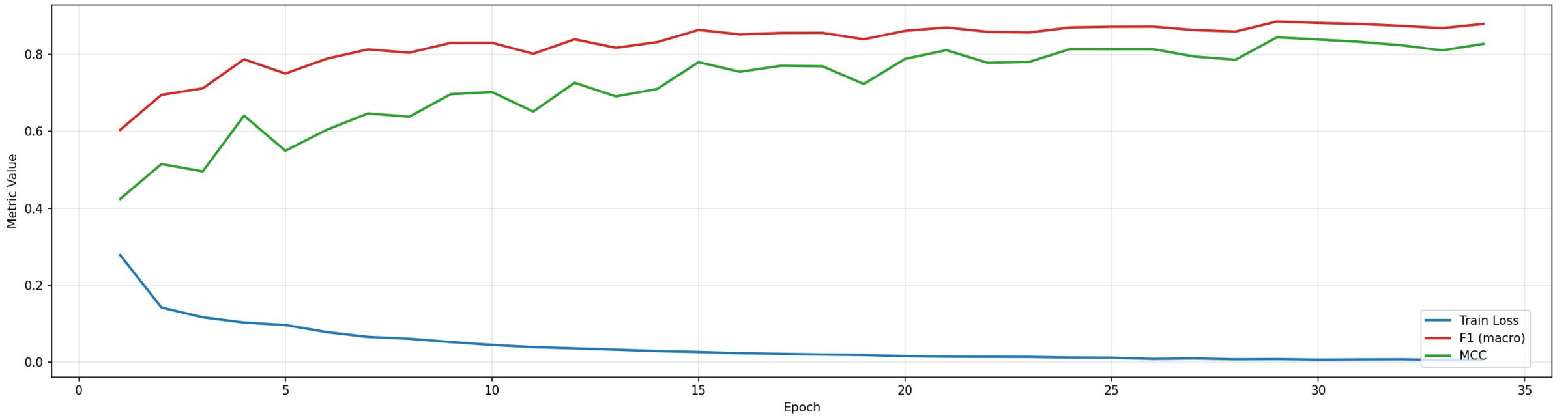**G**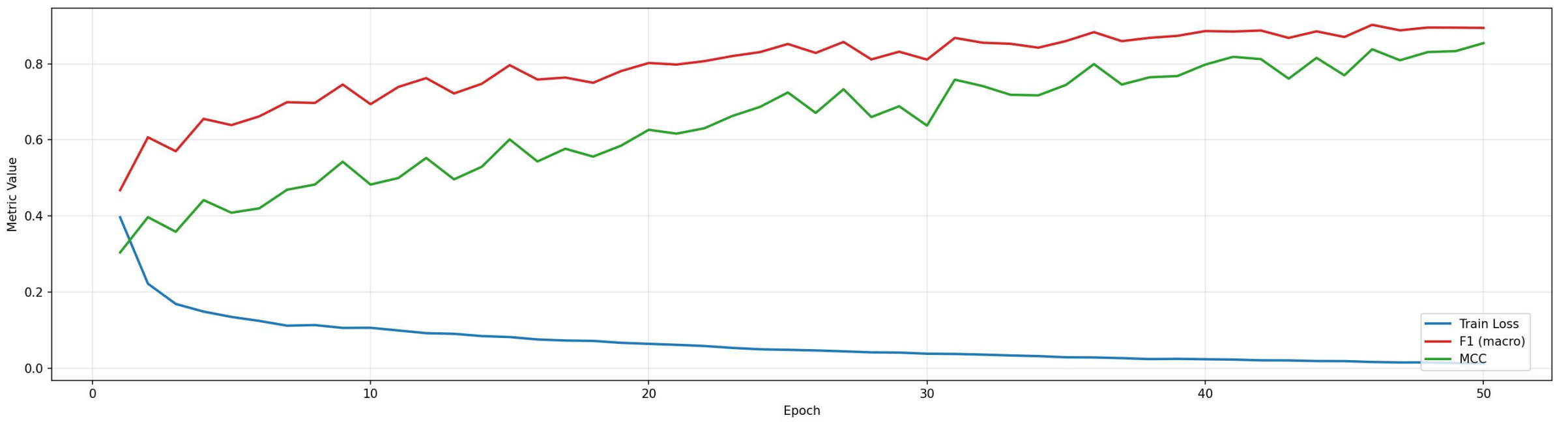**H**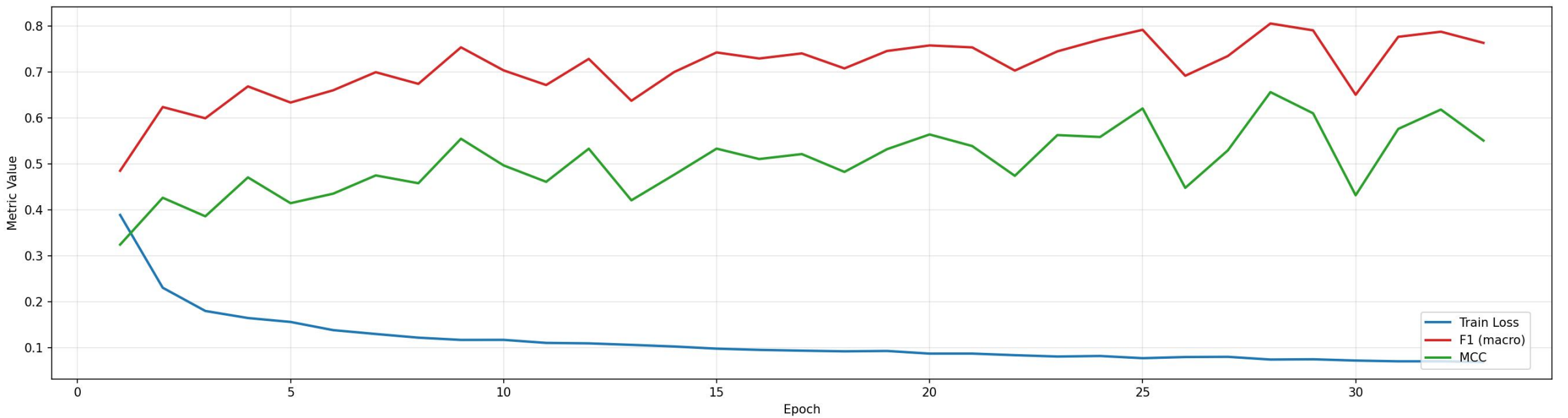

Supplement: Supplementary 1 — Figs. S1 to 15 Tables S1 to S8 [file csbj.0073.f1.zip › Fig_second_revision_S6.pdf]

**A**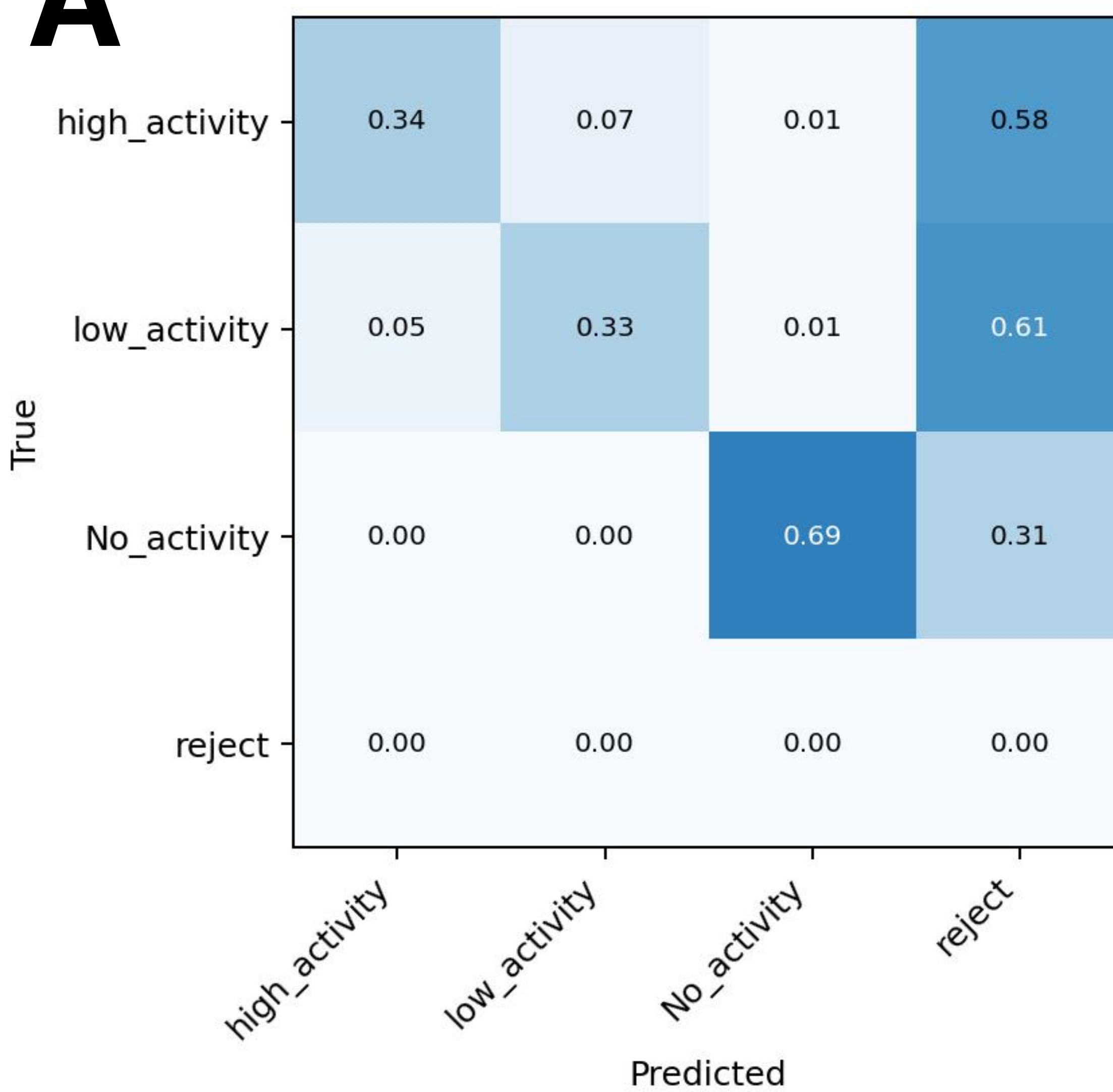**B**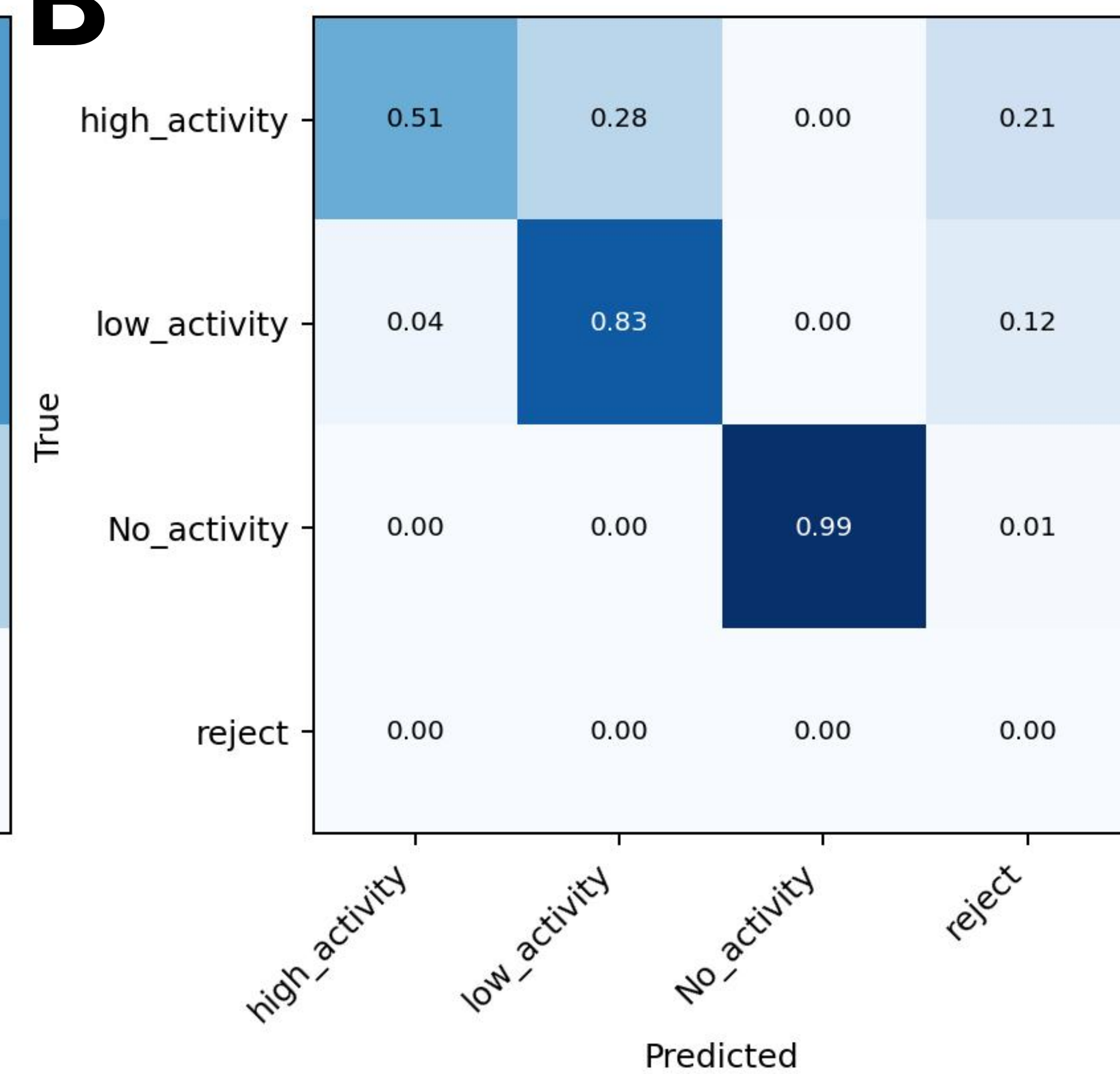**C**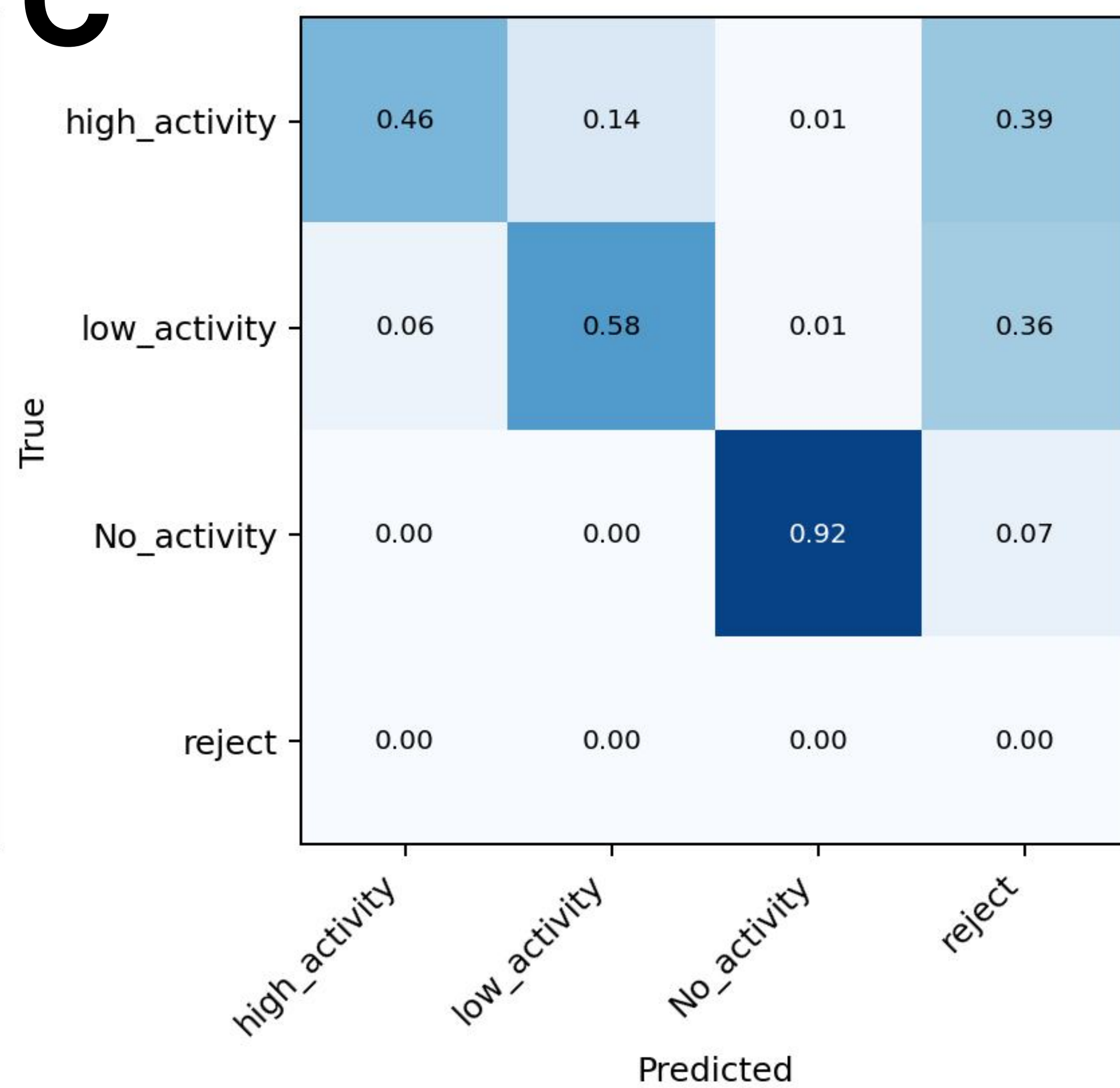**D**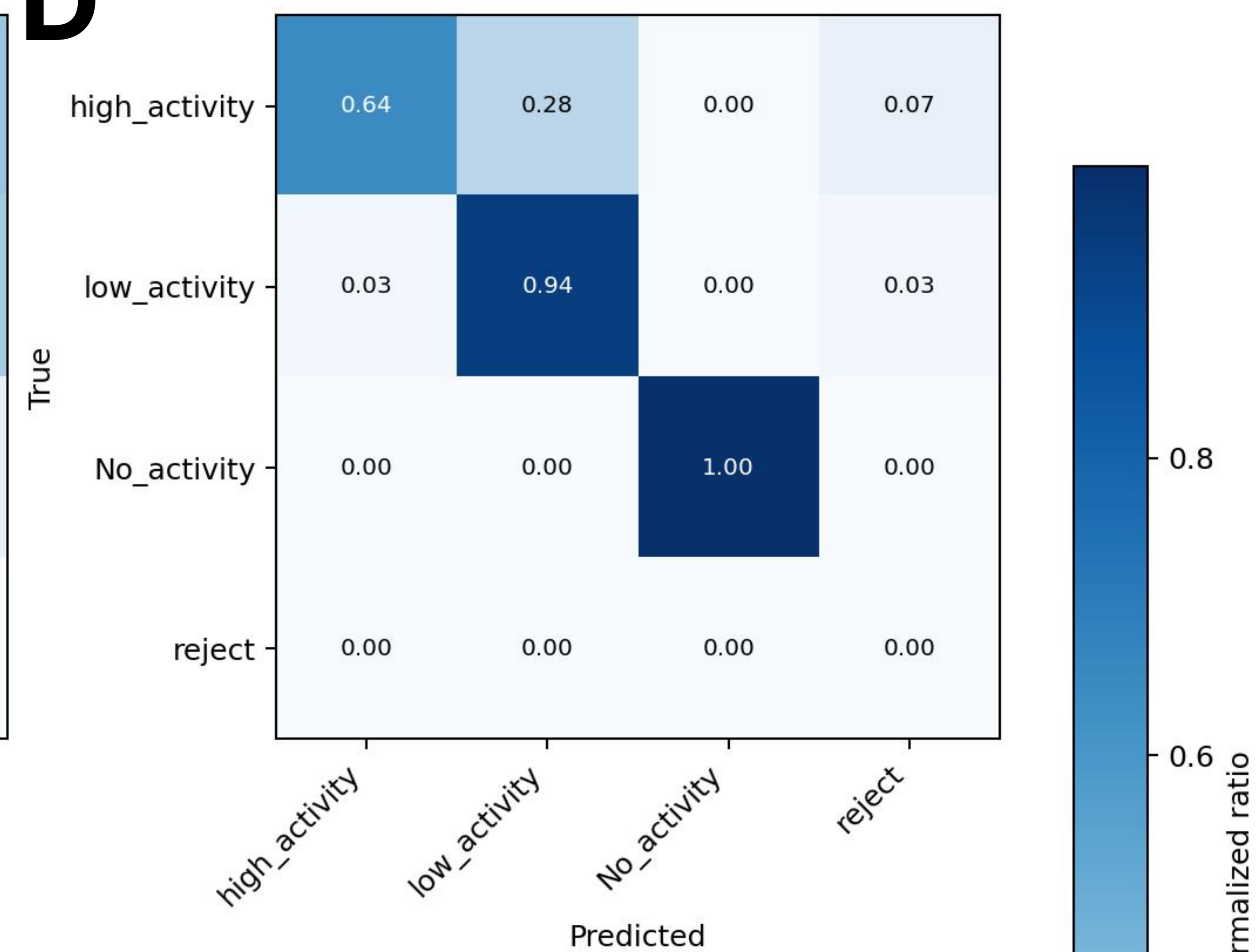**E**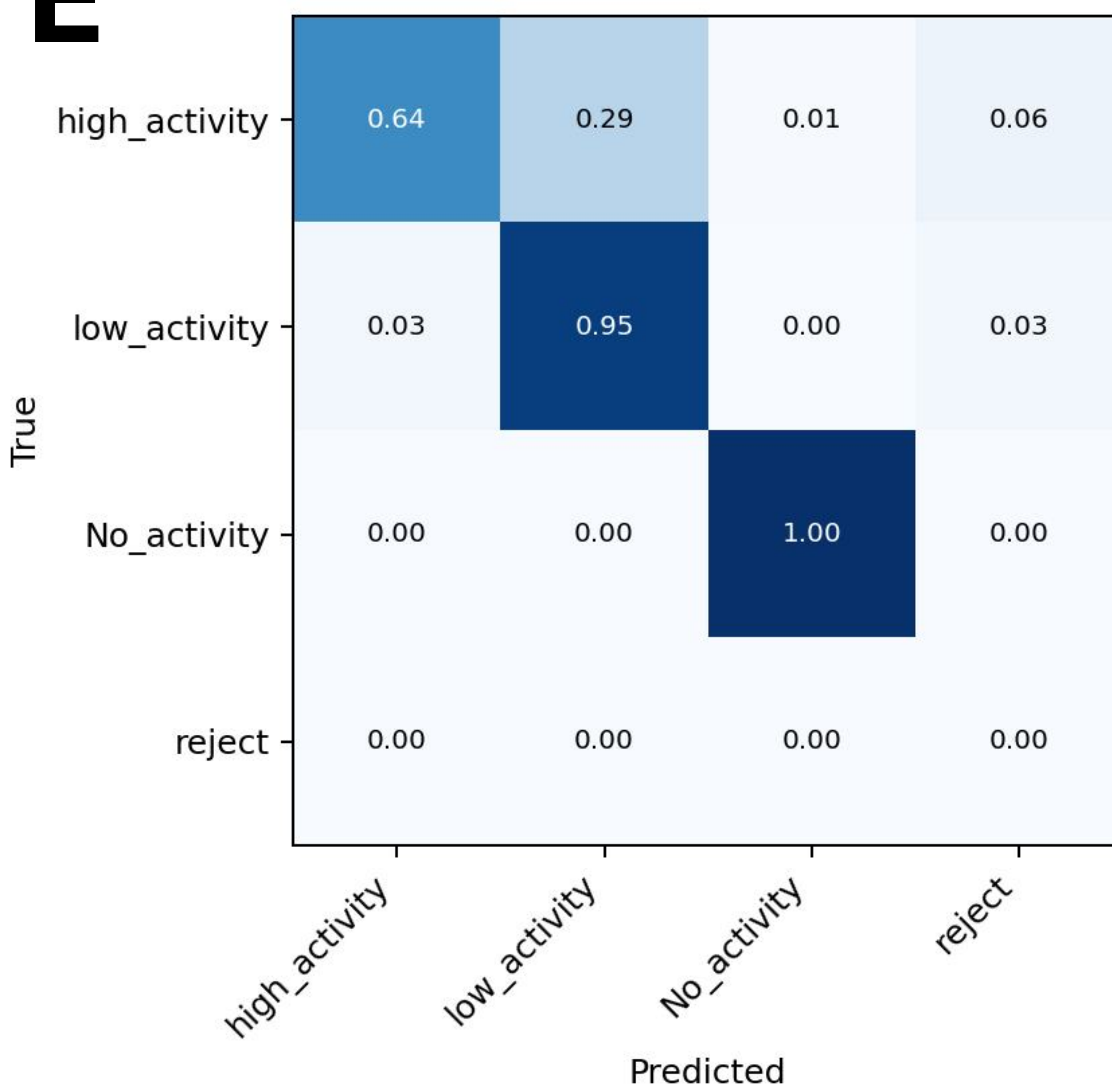**F**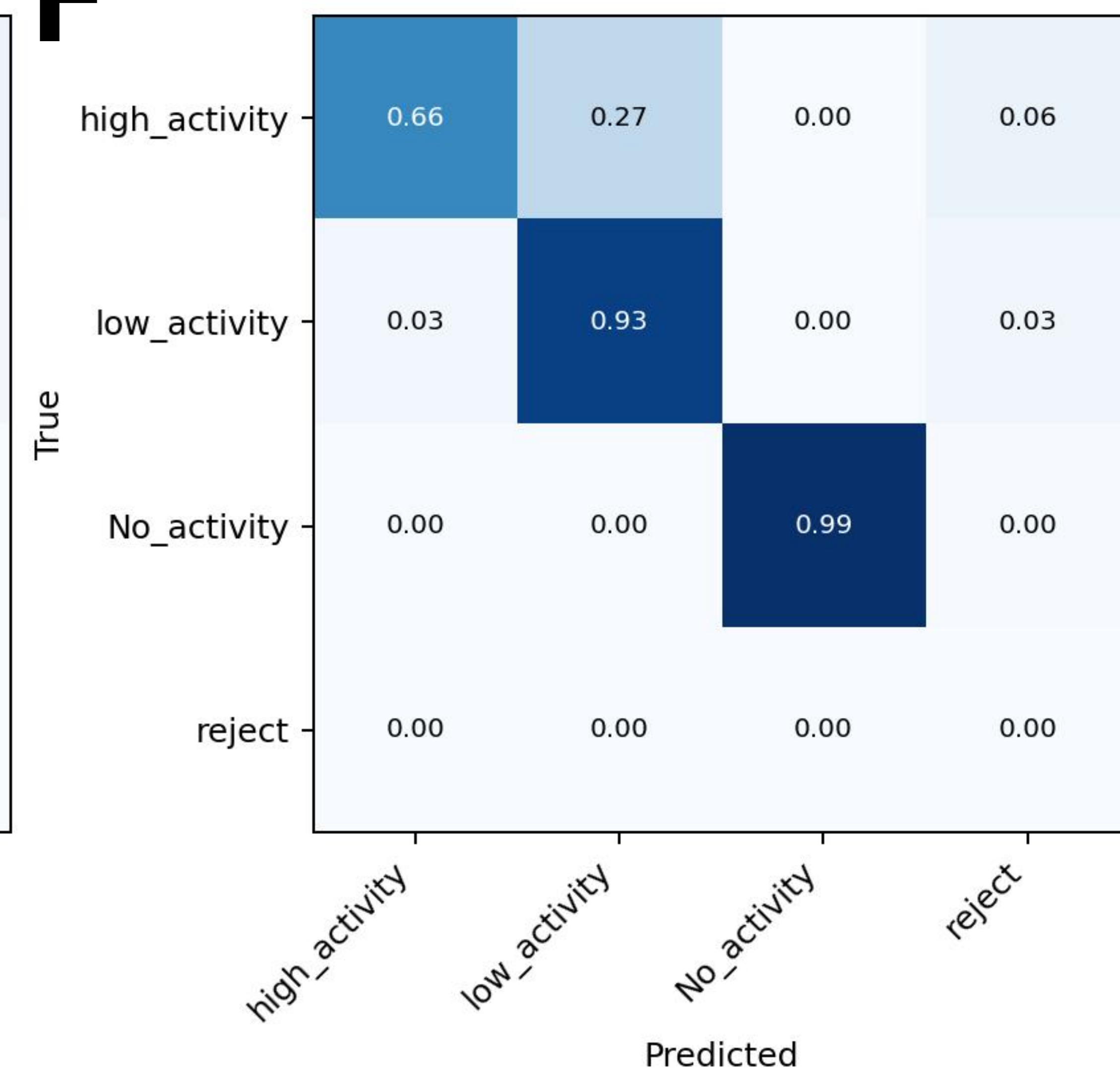**G**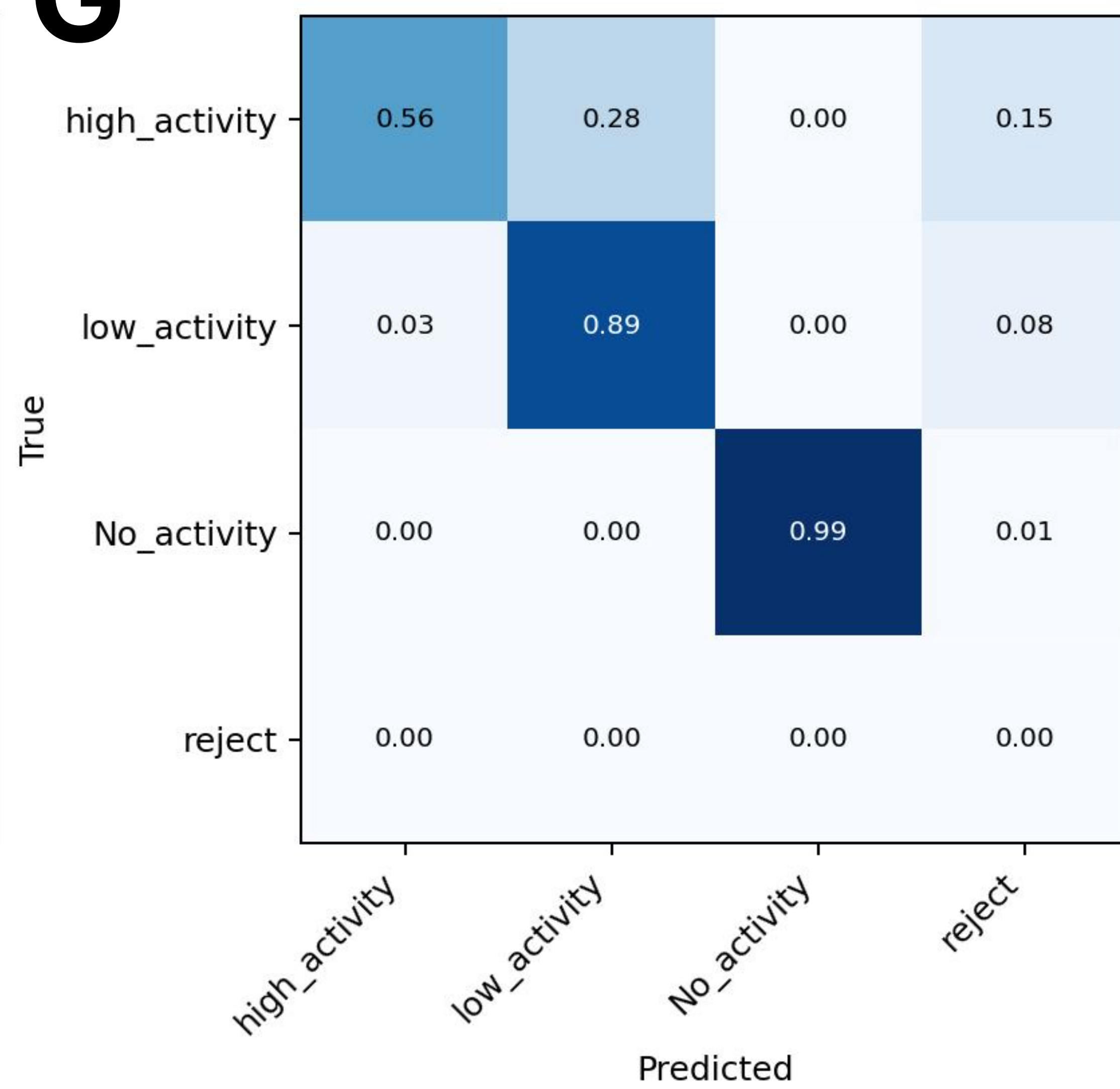**H**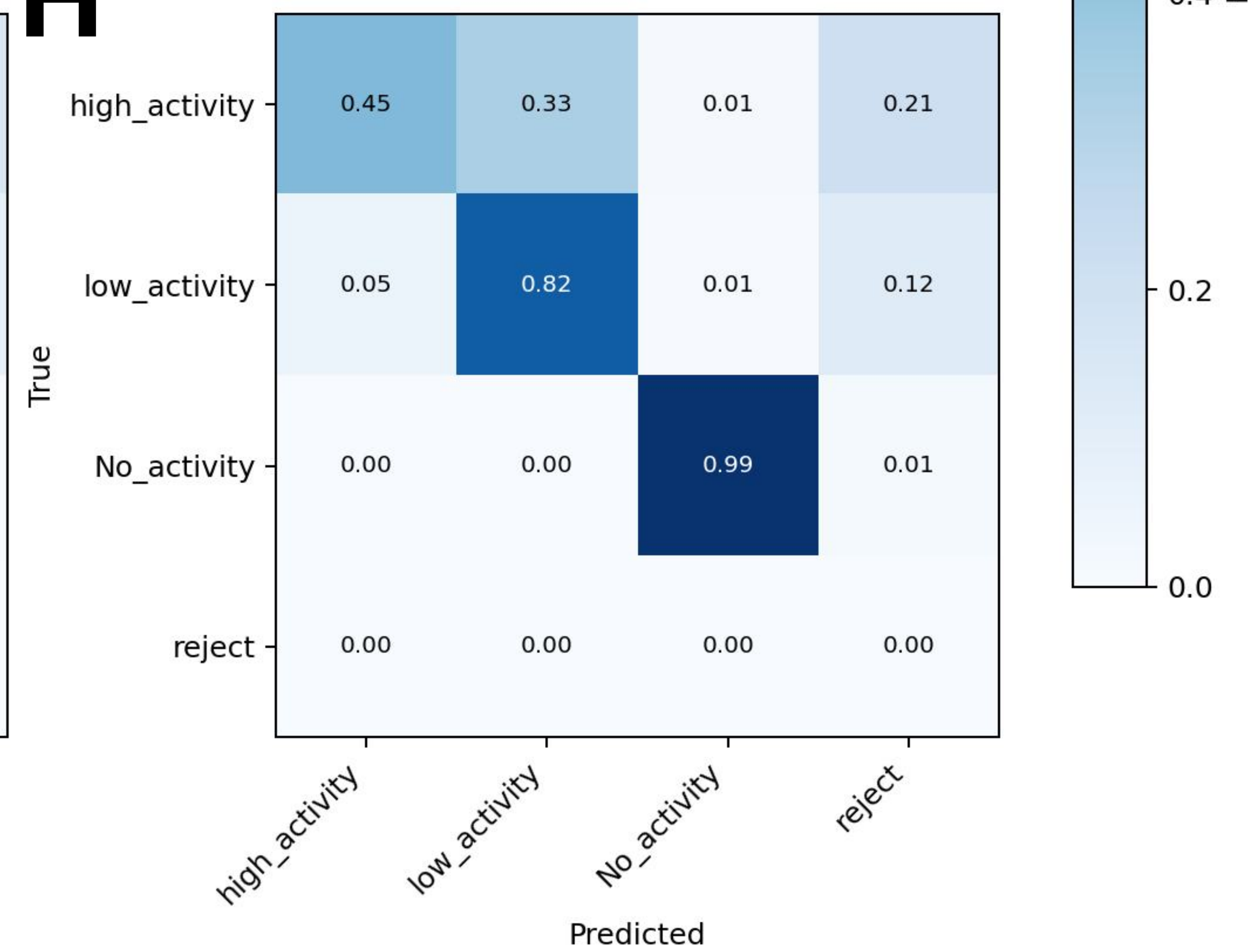

Supplement: Supplementary 1 — Figs. S1 to 15 Tables S1 to S8 [file csbj.0073.f1.zip › Fig_second_revision_S7.pdf]

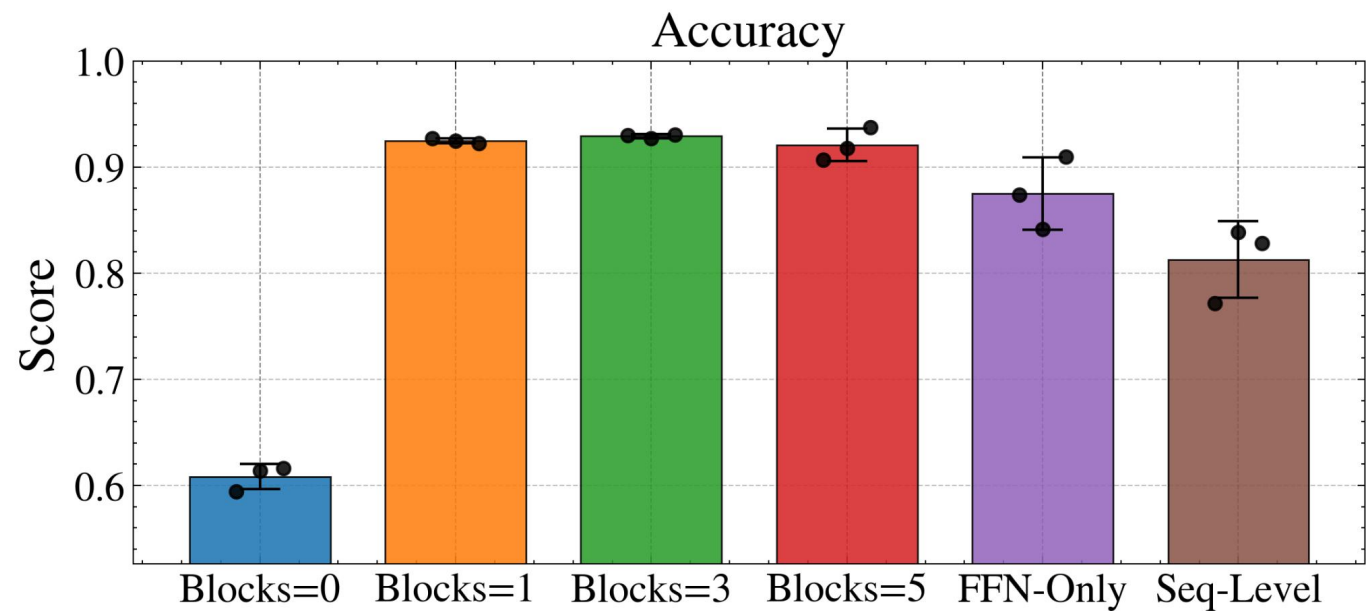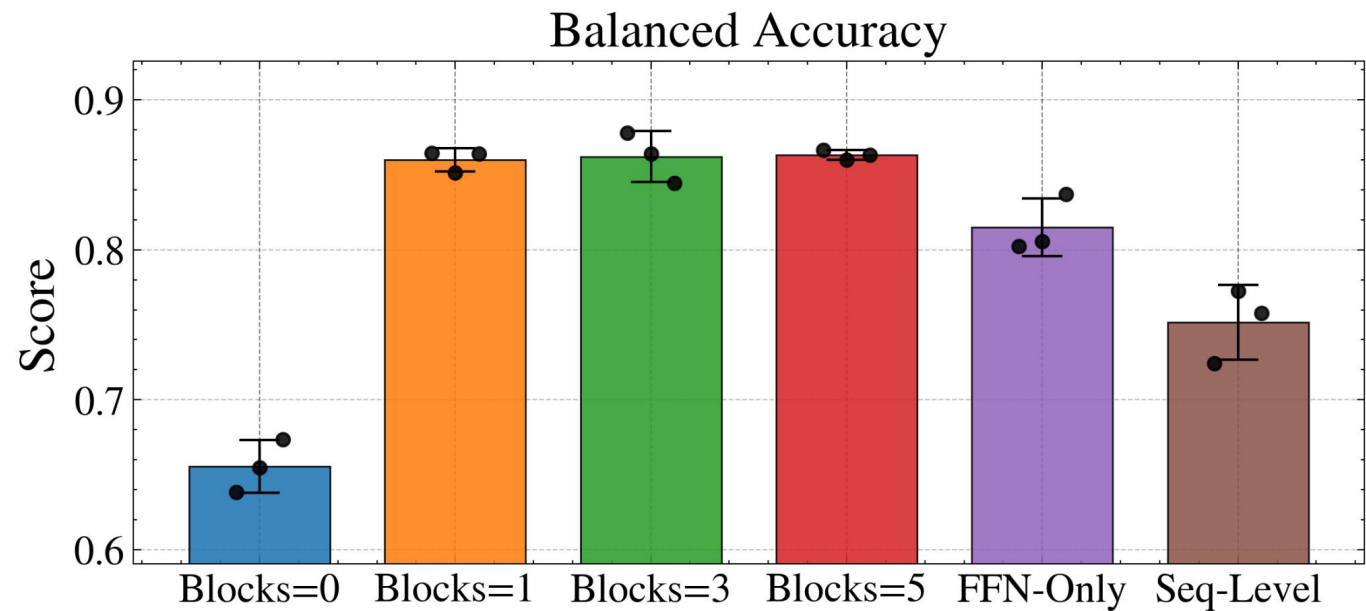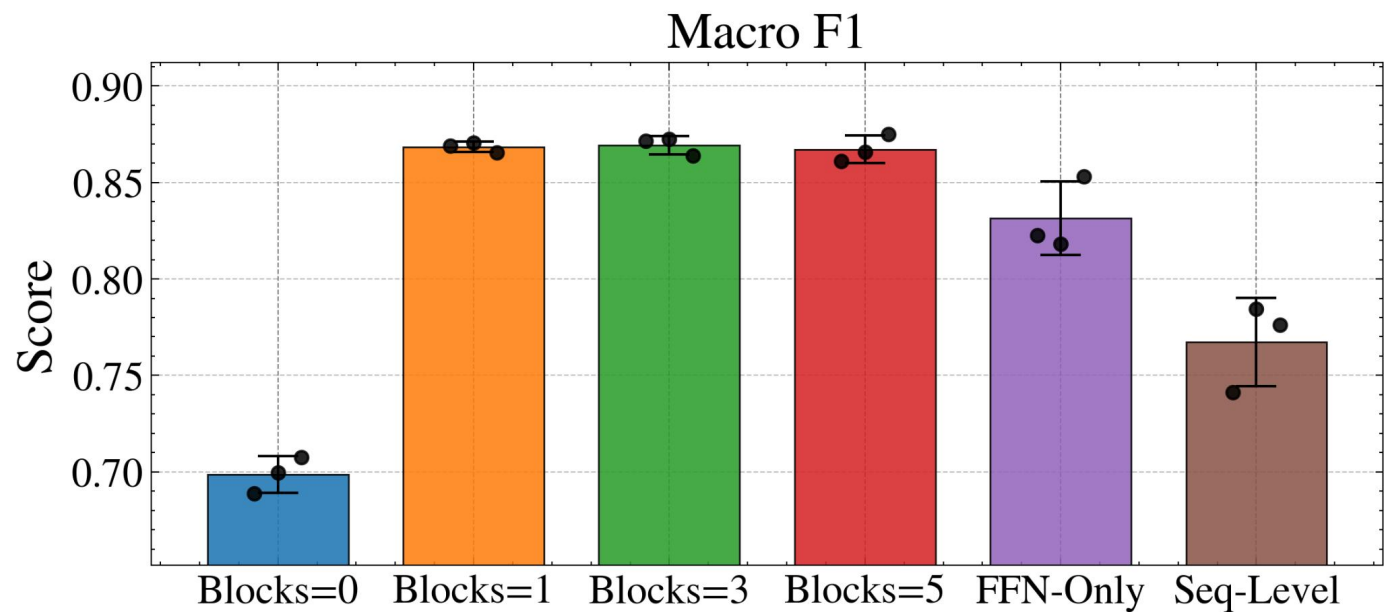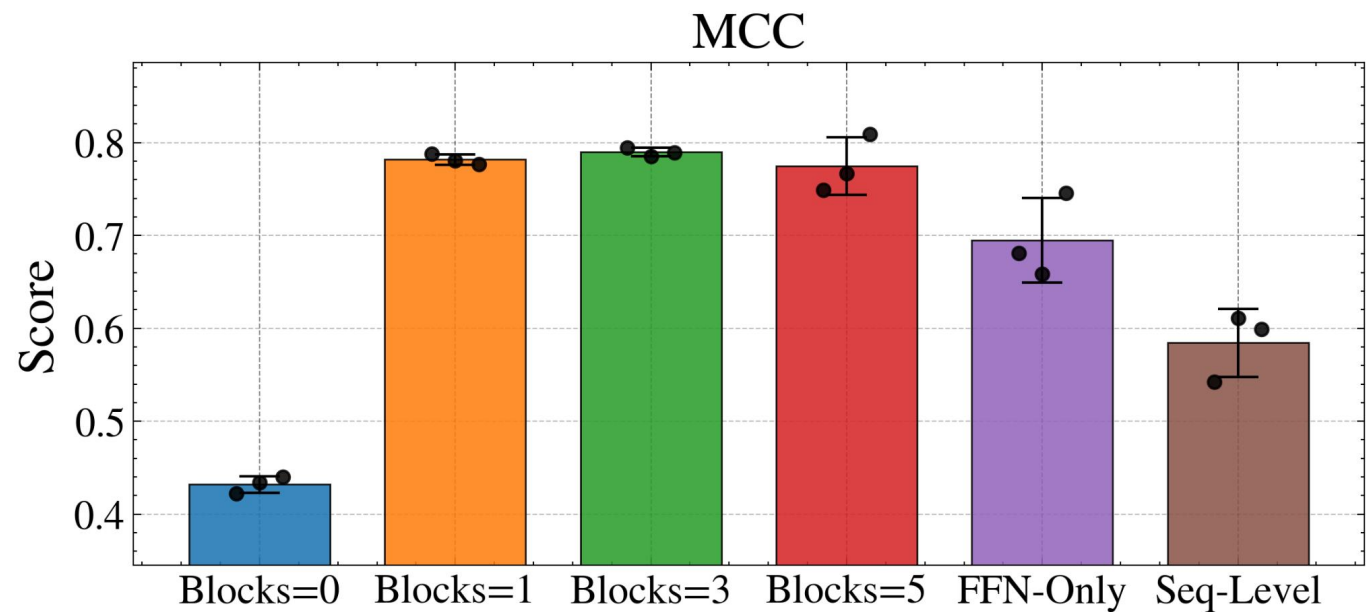

Supplement: Supplementary 1 — Figs. S1 to 15 Tables S1 to S8 [file csbj.0073.f1.zip › Fig_second_revision_S8.pdf]

**A**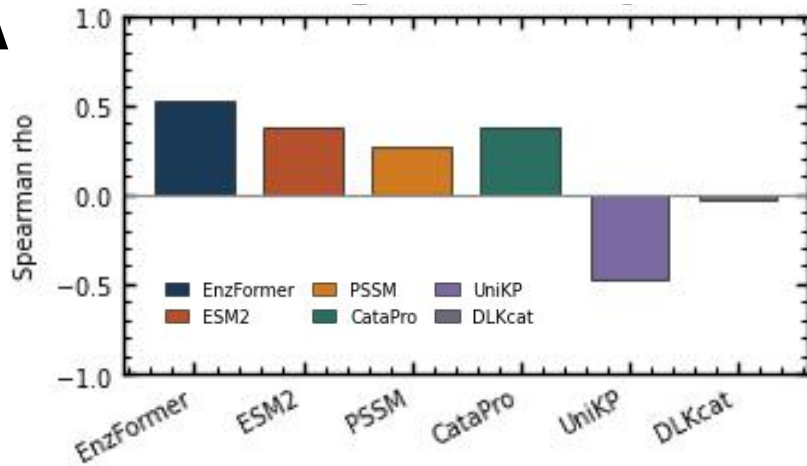**B**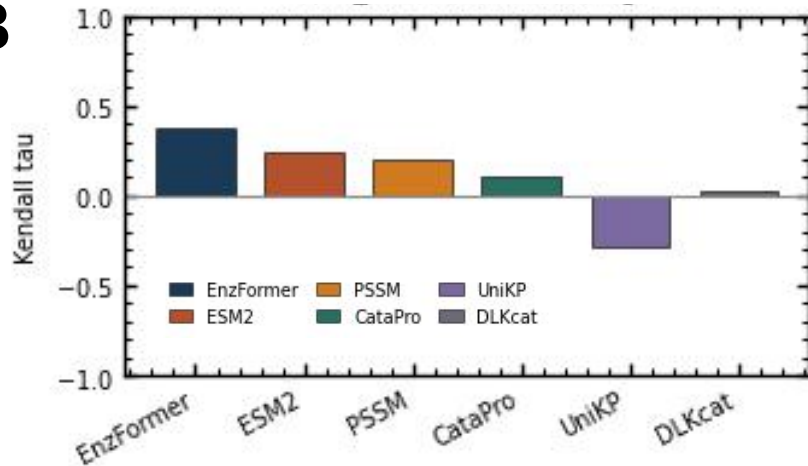

Supplement: Supplementary 1 — Figs. S1 to 15 Tables S1 to S8 [file csbj.0073.f1.zip › Fig_second_revision_S9.pdf]
